# Normalized Synergy Predicts That CD8 Co-Receptor Contribution to T Cell Receptor (TCR) and pMHC Binding Decreases As TCR Affinity Increases in Human Viral-Specific T Cells

**DOI:** 10.3389/fimmu.2017.00894

**Published:** 2017-07-28

**Authors:** Chad M. Williams, Alexandra A. Schonnesen, Shu-Qi Zhang, Ke-Yue Ma, Chenfeng He, Tori Yamamoto, S. Gail Eckhardt, Christopher A. Klebanoff, Ning Jiang

**Affiliations:** ^1^Department of Biomedical Engineering, University of Texas at Austin, Austin, TX, United States; ^2^McKetta Department of Chemical Engineering, University of Texas at Austin, Austin, TX, United States; ^3^Institute for Cell and Molecular Biology, University of Texas at Austin, Austin, TX, United States; ^4^Center for Cancer Research, National Cancer Institute, National Institutes of Health, Bethesda, MD, United States; ^5^Immunology Graduate Group, University of Pennsylvania, Philadelphia, PA, United States; ^6^LIVESTRONG Cancer Institutes, Dell Medical School, University of Texas at Austin, Austin, TX, United States; ^7^Center for Cell Engineering, Department of Medicine, Memorial Sloan Kettering Cancer Center (MSKCC), New York, NY, United States; ^8^Parker Institute for Cancer Immunotherapy, MSKCC, New York, NY, United States

**Keywords:** T cell receptor/pMHC interaction, two-dimensional affinity and kinetics, CD8 cooperation, CD8 binding independent T cell receptor, human viral-specific polyclonal CTL

## Abstract

The discovery of naturally occurring T cell receptors (TCRs) that confer specific, high-affinity recognition of pathogen and cancer-associated antigens remains a major goal in cellular immunotherapies. The contribution of the CD8 co-receptor to the interaction between the TCR and peptide-bound major histocompatibility complex (pMHC) has previously been correlated with the activation and responsiveness of CD8^+^ T cells. However, these studies have been limited to model systems of genetically engineered hybridoma TCRs or transgenic mouse TCRs against either a single epitope or an array of altered peptide ligands. CD8 contribution in a native human antigen-specific T cell response remains elusive. Here, using Hepatitis C Virus-specific precursor CTLs spanning a large range of TCR affinities, we discovered that the functional responsiveness of any given TCR correlated with the contribution of CD8 to TCR/pMHC binding. Furthermore, we found that CD8 contribution to TCR/pMHC binding in the two-dimensional (2D) system was more accurately reflected by normalized synergy (CD8 cooperation normalized by total TCR/pMHC bonds) rather than synergy (total CD8 cooperation) alone. While synergy showed an increasing trend with TCR affinity, normalized synergy was demonstrated to decrease with the increase of TCR affinity. Critically, normalized synergy was shown to correlate with CTL functionality and peptide sensitivity, corroborating three-dimensional (3D) analysis of CD8 contribution with respect to TCR affinity. In addition, we identified TCRs that were independent of CD8 for TCR/pMHC binding. Our results resolve the current discrepancy between 2D and 3D analysis on CD8 contribution to TCR/pMHC binding, and demonstrate that naturally occurring high-affinity TCRs are more capable of CD8-independent interactions that yield greater functional responsiveness even with CD8 blocking. Taken together, our data suggest that addition of the normalized synergy parameter to our previously established TCR discovery platform using 2D TCR affinity and sequence test would allow for selection of TCRs specific to any given antigen with the desirable attributes of high TCR affinity, CD8 co-receptor independence and functional superiority. Utilizing TCRs with less CD8 contribution could be beneficial for adoptive cell transfer immunotherapies using naturally occurring or genetically engineered T cells against viral or cancer-associated antigens.

## Introduction

The kinetic parameters that govern T cell activation, co-stimulation and functionality remain a point of discussion in the immunological field. The original attempts to quantify these parameters relied on surface plasmon resonance (SPR), a three-dimensional (3D) receptor–ligand interaction assay. SPR measures the number of immobilized ligands [peptide-bound major histocompatibility complexes (pMHC)] which bind to solubilized receptors [T cell receptor (TCR)] over time or *vice versa* to determine the rate constants that describe their binding and disassociation. Studies using this method have converged upon the 3D off-rate as the most accurate predictor of T cell cytolytic capacity (1–3). Despite this consensus, 3D measurement techniques fail to account for the geometric and physical constraints present in CTL-antigen presenting cell (APC) interactions (4–6). Two-dimensional (2D) techniques which take into account the complexities on the CTL surface have recently emerged and more accurately mimic CTL–APC interactions by either using micropipettes to impinge single CTLs upon membrane-bound pMHC (4, 7, 8), or by single molecule Förster resonance energy transfer (FRET) analysis of transfected blast T cells (6). Huppa et al. demonstrated with single molecule FRET imaging that the 2D on-rates and off-rates of TCR/pMHC interactions were significantly faster than previously accepted values in the 3D system, while the on-rate spanned a range of almost 50-fold in their transgenic TCR model. Using a micropipette adhesion assay, Huang et al. independently showed that 2D off-rate was faster than its 3D counterpart and a larger dynamic range of affinity were present in 2D compared to that of 3D, which was predominantly due to a wide range of on-rates and a small range of off-rates. They also found that 2D affinity and kinetic parameters correlated better with T cell proliferative response to peptide stimulation compared to their 3D counterparts (4).

The CD8 co-receptor contributes to TCR binding to pMHC by reducing the rate of dissociation between TCR/pMHC interaction (9). CD8 is present on the cell surface as αα homodimers or αβ heterodimers that associate with the TCR/pMHC complex (9, 10). On the MHC class one molecule, CD8 binds to the alpha 3 domain, distinctly separate from the TCR binding of the peptide, alpha 1 and alpha 2 domains (10). Several studies using either 2D (7, 11) or 3D kinetic measurement (9, 12, 13) techniques have shown that the binding affinity of CD8 to MHC is independent of TCR specificity or affinity, and the avidity of these three molecular interactions is larger than the simple addition of TCR/pMHC and CD8/pMHC interaction affinities. This inequality has driven the pursuit to interpret CD8 cooperation to TCR/pMHC binding. Previous studies have attempted to define this cooperation resulting from the binding of CD8, but a consensus between 2D and 3D studies has not been reached. Studies in the 3D system have shown that CD8 cooperation decreases with increased TCR affinity (14–16). A recent study using 2D kinetic measurement techniques suggested a positive correlation between CD8 cooperation (described as synergy) and TCR affinity, with CD8 cooperation increasing with TCR affinity (7). So far, studying CD8 cooperation has been limited to altered peptide ligands (APLs) (11, 15) or a few TCR transfected hybridoma cell lines (7). Thus, the CD8 cooperation present within naturally occurring, human, polyclonal precursor CTLs spanning a large dynamic range of 2D TCR affinity and kinetics remains unknown.

Recently, using a previously described tetramer enrichment strategy (8, 17, 18), we isolated a set of precursor CTLs from healthy HLA-A02:01 donors that recognize an HCV epitope (KLVALGINAV) (17). We then compared 2D TCR and CD8 binding of the HCV epitope in complex with HLA-A2:01 (referred to as pMHC throughout) with antigen-specific CD8^+^ T cell clones spanning several logs of binding affinity. These CTL clones allowed us to comprehensively examine the 2D affinity and 2D kinetic constants for expanded polyclonal human viral precursor TCRs (8). As had been demonstrated in APL and mouse TCR transgenic hybridomas (7, 11, 19), the 2D micropipette adhesion assay enabled the interrogation of co-receptor molecules on the CTL’s surface. We propose that our library of viral-specific polyclonal CTLs could be used to extend the understanding of the kinetics of co-receptor molecules on the surface of primary human CTLs. First, we showed that a dynamic range of roughly three orders of magnitude in affinity was present in virally specific CD8^+^ T cells and that this affinity range corresponds to an equally diverse on-rate range. Within this dynamic affinity range, the CD8 contribution to TCR/pMHC binding inversely correlates with peptide sensitivity and positively with granular release. Furthermore, we demonstrated the presence of two high-affinity TCRs that require only minimal CD8 contribution in binding cognate peptide–MHC complexes.

Importantly, CD8-independent TCR expressing CTLs were capable of functional response even when most of CD8 molecules were blocked during cognate peptide stimulation. By contrast, CD8-dependent TCR expressing CTLs were shown to completely lose functional responses when about 20% of CD8 were blocked. Previous studies have demonstrated that the CD8^+^ T cells in the peripheral blood exhibit varying levels of CD8 expression (20–22). CD8^+^ T cells that express lower levels of CD8 have been shown to be functionally distinct from CD8^+^ T cells expressing high levels of CD8 (20), and in comparison to CD8^+^ T cells expressing high levels of CD8, elicit poor cytotoxic capabilities (21, 22). Thus, the CD8-independent TCRs we identified this way should be ideal in maintaining functionality despite varying degrees of CD8 expression levels, making them ideal for TCR re-directed adoptive cell therapy.

## Materials and Methods

### MHC Monomers, Peptide, and MHC Tetramers Used

Ultraviolet (UV)-cleavable peptide-loaded biotinylated pMHC-CD8wt and pMHC-CD8mut monomers were obtained from the National Institutes of Health Tetramer Core Facility. HCV (KLVALGINAV), preproinsulin (PPI, ALWMRLLPL), and NY-ESO-1 (SLLMWITQV) peptides of 95% purity were synthesized (Genscript) and loaded onto both pMHC-CD8wt and pMHC-CD8mut (with D227K/T228A mutations that abrogate CD8 binding) monomers using UV light as previously described (17, 23). Monomers were conjugated to allophycocyanin-labeled streptavidin (Biolegend) to create tetramers to identify HCV-specific T cells.

### HCV-Specific CD8^+^ T Cell Sorting and Isolation from Human Blood Donors

HCV-specific T cells from HCV seronegative donors were isolated as previously described (8, 17, 18). Human leukocyte reduction system chambers were obtained from de-identified donors with strict adherence to guidelines from the University of Texas at Austin Institutional Review Board. CD8^+^ T cell enrichment was performed as previously described (8, 17, 18) using tetramer. CD8^+^ T cells were isolated by the following series of steps: first, non-CD8^+^ T cells and non-viable cells were gated out by using a negative selection strategy of events positive for CD4, CD14, CD16, CD19, CD32, and CD56. Then naïve HCV-specific CD8^+^ T cells were sorted based on HCV tetramer^+^ and CD45RA^+^CCR7^+^CD27^+^ phenotype as single cells into medium for culture (8).

### TCR, CD8α, and CD8β Surface Expression Analysis

T cell receptor, CD8α, CD8β, and pMHC surface expression analyses were performed as previously described using PE-conjugated antibodies, and PE-labeled standardized beads (quantibrite beads, BD biosciences) (11). For TCR, CD8α, CD8 β, and pMHC the following PE-conjugated antibodies were used: clone IP26, Biolegend; clone SK1, Biolegend; clone 2ST8.5H7, Beckman Coulter; and clone BB7.2, Biolegend.

### Immobilize MHC Monomers to Red Blood Cells (RBCs)

Biotinylated RBCs were conjugated with streptavidin and peptide exchanged biotinlyated MHC monomers following previously described protocols (4, 8, 11). HCV clones, pMHC-CD8wt, and pMHC-CD8mut-coated RBCs were added to the microscope chamber and TCR + CD8, TCR, and CD8 kinetics interrogated as previously described (4, 11, 19). To measure TCR/CD8/pMHC, TCR/pMHC, and CD8/pMHC interaction pMHC-CD8wt-HCV, pMHC-CD8mut-HCV, and PPI peptide in complex with pMHC-CD8wt monomer-coated RBCs were used. To quantify non-specific interaction, the non-specific peptide PPI was loaded into pMHC-CD8mut monomer, and contacted with each HCV clone similarly to other 2D micropipette interactions.

### 2D TCR Affinity, 2D On- and Off-Rate Calculation

Calculation of kinetic parameters such as 2D affinity, on-rate, and off-rate for TCR/pMHC and CD8/pMHC interactions were performed at both room temperature (main figures) and 37°C (Figures S9 and S10 in Supplementary Material) as previously described (4, 19). The micropipette adhesion frequency assay utilizes micromanipulation technology in combination with piezo actuators to bring pMHC-conjugated RBCs in contact with TCRs on the surface of CTLs or primary cells isolated using *in situ* TCR affinity and sequence test (iTAST) (8). Adhesion events are measured visually with an adhesion event being recorded as 1, while a no adhesion event recorded as 0, and adhesion frequency, *P*_a_, being calculated by dividing number of adhesion events by total contacts between RBC and T cell. As described previously, the adhesion curve can be fit with a known model for reversible bimolecular interaction at 2D surfaces according to the following equation:

(1) $$P_{a}=1-\text{exp}\left[-m_{\text{TCR or CD8}}m_{\text{pMHC}}A_{c}K_{a}\left(-k_{\text{off}}t_{c}\right)\right].$$

Site densities of TCR, CD8, and pMHC are denoted as *m*_TCR_ or *m*_CD8_, and *m*_pMHC_, respectively, with *K*_a_, *k*_off_, and *t*_c_ representing 2D affinity, 2D off-rate, and contact time. *A*_c_ is the contact area with the constant radius of 1 µm. The 2D affinity measured in this way uses a product of *A*_c_*K*_a_, which has a unit of μm^4^. Using this equation as a model, we used experimental values of TCR or CD8 and pMHC site densities in combination with *P*_a_ at varying contact times to obtain the best estimate of 2D affinity and off-rate using Chi-squared curve fitting. Alternatively, 2D affinity (*A*_c_*K*_a_) can be estimated from the equilibrium phase of the adhesion curve (see Eq. 3 below). In addition, 2D affinity is the ratio between the 2D on-rate, *k*_on_, and 2D off-rate, *k*_off_. Thus, the 2D on-rate can be calculated by the following equation:

(2) $$A_{c}k_{\text{on}}=k_{\text{off}}\times A_{c}K_{a}.$$

To calculate 2D affinity from the equilibrium phase of adhesion curve, a log transformation of Eq. 1 results in *A*_c_*K*_a_ with a relationship with adhesion probability *P*_a_ at equilibrium (eq), as *t_c_*→∞, which simplifies Eq. 1 to the following equation:

(3) $$A_{c}K_{a}=-ln\left[1-P_{a}\left(\text{eq}\right)\right]/\left(m_{\text{TCR or CD8}}\times m_{\text{pMHC}}\right).$$

TCR/CD8/pMHC, CD8/pMHC, and TCR/pMHC kinetics were shown to come to equilibrium at 4 s of contact time. Therefore, adhesion frequency obtained at 4 s of contact time was used to calculate number of bonds formed per pMHC for TCR/pMHC and CD8/pMHC bimolecular as well as TCR/CD8/pMHC tri-molecular interaction using the following equation:

(4) $$\langle n\rangle /m_{\text{pMHC}}=-\text{ln}\left[1-P_{a}\left(\text{eq}\right)\right]/m_{\text{pMHC}}.$$

### Synergy and Normalized Synergy Calculation

To estimate the contribution of CD8 to total receptor binding to pMHC, we used the previously described parameter synergy (7) as denoted by equation:

(5) $$\text{synergy}=\frac{\langle n_{\text{TCR}+\text{CD8}}\rangle }{m_{\text{pMHC}-\text{CD8wt}-\text{HCV}}}-\frac{\langle n_{\text{TCR}}\rangle }{m_{\text{pMHC}-\text{CD8mut}-\text{HCV}}}-\frac{\langle n_{\text{CD8}}\rangle }{m_{\text{pMHC}-\text{CD8wt}-\text{PPI}}}.$$

Equation 5 calculates the contribution of CD8 to TCR/pMHC interaction by subtracting total number of bonds formed per pMHC mediated by either TCR/pMHC or CD8/pMHC bimolecular interactions from (TCR + CD8)/pMHC tri-molecular interactions, which results in total number of bonds per pMHC due to CD8 cooperation. Normalized synergy takes synergy (Eq. 5) and divides it further by total number of TCR/pMHC bonds formed per pMHC as shown in following equation:

(6) $$\text{normalized synergy}=\frac{\text{Synergy}}{\left[\frac{\langle n_{\text{TCR}}\rangle }{m_{\text{pMHC}-\text{CD8mut}-\text{HCV}}}\right]}.$$

### CD107a Mobilization, Peptide Sensitivity, and Complete CD8 Blocking Assays

B cell lymphoma JY cell line expressing HLA-A02:01 was used as target cells in a 2:1 ratio with HCV clones. Duplicates were performed for all stimulation conditions, with all stimulation conditions taking place for 4 h at 37°C. 100 µL of culture medium containing monensin (Biolegend), CD107a antibody (Biolegend), with 50,000 HCV clones and 100,000 JY cell remained the same throughout all conditions. HCV peptide solubilized in DMSO was titrated in culture medium, maintaining less than 1% DMSO by volume. Various concentrations of HCV peptide (50 µM–1 pM) were present throughout the incubation as previously described (17, 24). All HCV peptide stimulation experiments were performed as duplicates and irrelevant peptide NY-ESO-1 was used as a negative control at 1 µM. At the end of incubation, cells were washed and fluorescence intensity of CD107a was read for T cells. Percentage of CD107a positive cells due to irrelevant peptide was subtracted from percentage of CD107a positive cells stimulated by various concentrations of HCV peptide. CTL functionality when CD8 was completely blocked was performed by using CD8 blocking antibody, which was previously described to best block CD8 in tetramer staining in human CTL clones (FITC-labeled clone DK25, EMD Millipore) (25), in cell media throughout the 4 h CD107a assay at 2.5 µg/mL. Phorbol 12-myristate 13-acetate (PMA) and ionomycin (eBioscience) stimulation was performed as per manufacturer instructions.

### CD107a Mobilization with CD8 Blocking Antibody Titration Assay

For CD107a experiment with CD8 blocking antibody titration, 50,000 T cells for each CTL clone were stained with varying concentrations (2.5–0.0005 µg/mL) of CD8 blocking antibody (FITC-labeled clone DK25, EMD Millipore) (25) for 25 min prior to the addition of cell media with 10 µM HCV peptide and 100,000 JY cells for CD107a stimulation. The CD8 blocking antibody was present throughout 4-h stimulation period. After 4 h of stimulation, cells in each well were split into two aliquots. Half of cells were stained with additional saturating concentration (2.5 µg/mL) of CD8 blocking antibody (FITC-labeled clone DK25, EMD Millipore) to measure the MFI of total CD8 expression. The MFI of the CD8 blocking antibody were directly read from the other half of cells without adding additional CD8 blocking antibody to assess the MFI of CD8 that was blocked during stimulation (Figure S6 in Supplementary Material). The MFI ratio between the CD8 blocking antibody read from these two aliquots represents the percent of CD8 molecules being blocked at various titrations of CD8 blocking antibody during the stimulation (Figure S6 in Supplementary Material). Irrelevant peptide stimulation, NY-ESO-1, was performed in a similar manner with a fixed 10 µM peptide. Percentage of CD107a positive T cells from irrelevant peptide stimulation was subtracted off from that of 10 µM HCV peptide stimulation. If resulted percentages of CD107a were less than 1% after irrelevant peptide stimulation subtraction, then CTLs were considered as non-functional at this peptide concentration. CD8 blocking antibody titration was performed in a single well for each CTL clone with a repeat experiments performed on a different day.

### Intracellular Cytokine Staining

HCV peptide stimulation was performed as previously described (26–28). 50,000 T cells from each CTL clone was stimulated with 10 µM HCV peptide, or 10 µM HCV peptide with saturating concentration (2.5 µg/mL) of CD8 blocking antibody, or 10 µM of irrelevant peptide, NY-ESO-1, or PMA + ionomycin in cell media at 37°C for 5 h in the presence of brefeldin A and monensin to inhibit release of cytokine upon T cell activation. After 5 h, cells were fixed and permeabilized, then stained with anti-human IL-2, TNF-α, and IFN-γ specific antibodies for 30 min. Stained cells were washed twice and analyzed by flow cytometry. 10 µM HCV peptide with CD8 blocking antibody condition was only stained with TNF-α and IFN-γ-specific antibodies since both IL-2 and CD8 blocking antibody were conjugated with FITC.

### Statistical Analysis

Spearman rank correlation was used to determine correlation between parameters, with a *p*-value less than 0.05 considered as statistically significant. One-way ANOVA was performed to determine statistical differences between means, with a *p*-value less than 0.05 considered statistically significant. Chi-squared curve fitting was used in combination with the adhesion frequency and surface expression data obtained from TCR/pMHC and CD8/pMHC interaction kinetics to interpolate 2D TCR and CD8 on-rate and off-rates (Figure S1 in Supplementary Material). Remaining information regarding statistical analysis was denoted in figure captions.

## Results

### Similar to 2D Affinity, 2D TCR On-Rate Spans a 500-Fold Range for Antigen-Specific Polyclonal Human T Cells

Two-dimensional affinity and kinetics have been shown to better correlate with T cell function compared with 3D affinity. However, many studies, thus, far have been limited to using transgenic mouse TCRs against a set of APLs with different affinities and TCR stimulatory capacities. The use of APLs against a transgenic TCR has helped to elucidate many aspects of T cell functional responsiveness and TCR activation (3, 29–35), yet it does not adequately address the range of TCR/pMHC binding conformations, differential TCR signaling capacities, and cellular variation that would exist in a native polyclonal T cell response to a single antigen. Thus, the dynamic range of 2D TCR affinity and kinetics in the context of a human polyclonal T cell response to a single viral antigen has not been previously evaluated. Using a tetramer enrichment strategy described previously (17, 18), we isolated a set of precursor CTLs that recognize human a single HCV epitope in complex with pMHC (here denoted as pMHC-CD8wt-HCV) from healthy HCV seronegative blood donors (8). These precursor CTL clones allowed us to comprehensively examine the 2D affinity and 2D kinetic rate constants for viral antigen-specific polyclonal human TCRs.

**Figure 1:**
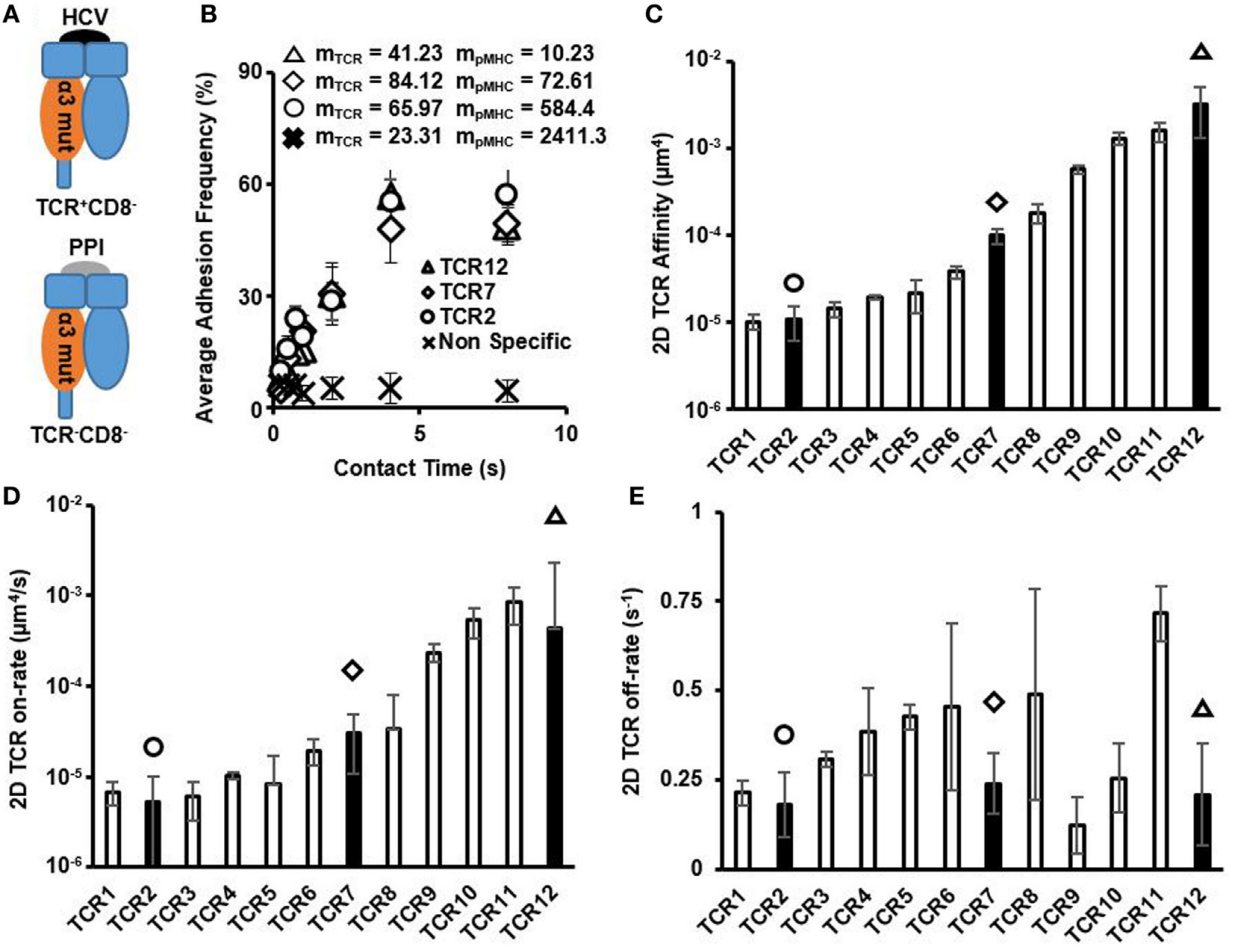
Two-dimensional (2D) T cell receptor (TCR)/pMHC affinity and kinetics of HCV-specific CD8+ T cell clones. **(A)** Schematics of different peptides and MHC mutants used to measure TCR/pMHC interaction and non-specific interaction control. **(B)** Average adhesion frequency for three examples of 2D TCR membrane kinetics of functional HCV-specific precursor CTLs. Non-specific adhesion was measured on TCR12 by quantifying TCR interaction with red blood cell conjugated with pMHC-CD8mut-preproinsulin (PPI) at same contact times as used in 2D TCR affinity measurements. Values of m_TCR_ and m_pMHC_ are the TCR and pMHC site density (molecules/μm2) for each respective CTL when measurement was performed. **(C)** 2D TCR affinity was calculated using Eq. 3, in Section “Materials and Methods,” for all functional HCV-specific CTLs and were depicted in order of ascending TCR affinity. Filled bars represent the 2D TCR affinity of the three example TCRs in **(B)** with matching symbols. **(D)** 2D TCR on-rate in order of ascending TCR affinity. 2D TCR on-rate was calculated using Eq. 2, in Section “Materials and Methods,” and TCR off-rate. Filled bars with symbols indicating individual TCRs depicted in **(B)**. **(E)** 2D TCR off-rate in order of ascending TCR affinity. 2D TCR off-rate was calculated using Chi-squared curve fitting of the 2D TCR membrane kinetic curves for each CTL, respectively. Filled bars denoting TCRs depicted in **(B)**. All data shown as mean ± SD of at least three cell pairs.

Using a pMHC mutant that abrogated CD8/pMHC in HCV-specific CTLs (here denoted as pMHC-CD8mut-HCV), we distinguished TCR/pMHC interactions from CD8/pMHC interaction (Figure 1A; Figures S1 and S2A,B in Supplementary Material). These interactions were specific to the HCV peptide as switching ligand to an irrelevant PPI peptide in complex with pMHC-CD8mut (here denoted as pMHC-CD8mut-PPI) resulted in background level of non-specific adhesion (Figure 1B). Micropipette adhesion frequency assay interrogates 2D affinity and kinetics by measuring the gradual increase of adhesion frequency between TCRs and pMHCs as a function of contact duration between TCRs on a T cell and pMHCs coated on a RBC with their respective surface densities. As such, the three example TCRs with progressively higher affinities had similar adhesion frequency curves, but the ligand site density differed about 7, 8, and 50-fold (Figure 1B). These combinations, therefore, yielded orders of magnitude differences on 2D affinity (Figure 1C). However, their adhesion frequency curves approached saturation at similar rates (Figure 1B), which produced similar 2D off-rates among these TCRs (Figure 1E). There was no apparent correlation between 2D affinity and 2D off-rate (Figure S3 in Supplementary Material). 2D affinity differed by orders of magnitude and that in combination with small differences on 2D off-rate resulted in 2D on-rate that varied by orders of magnitude (Figure 1D). This large dynamic range of 2D affinity and 2D on-rate is similar to what has been measured using mouse TCRs interacting with various APLs using a similar micropipette adhesion test (4) or single molecular FRET (6). It is worth noting that some of the high-affinity TCRs reported here are higher in affinity compared to commonly recognized high-affinity murine TCR, OTI, which has an affinity of 2.4 × 10^−4^ (μm^4^) as measured by micropipette adhesion test (4). In addition, 2D TCR affinities for these CTL clones measured at 37°C were all higher than that from room temperature, however, did not change the relative order of these clones in terms of affinity (Figure S9 in Supplementary Material), which is similar to what has been shown before for mouse TCR (4). Collectively, the large dynamic range that each TCR had toward slightly different ligands in combination with an equally large dynamic range of a polyclonal precursor TCRs toward the same ligand suggests that the possible antigen space the TCR repertoire could cover may exponentially increase depending on the TCR diversity of an organism. This also suggests a large degree of TCR cross-reactivity is inevitable (32).

### Human CD8/pMHC Affinity Is Orders of Magnitude Smaller Compared to Strong TCR/pMHC Interaction

**Figure 2:**
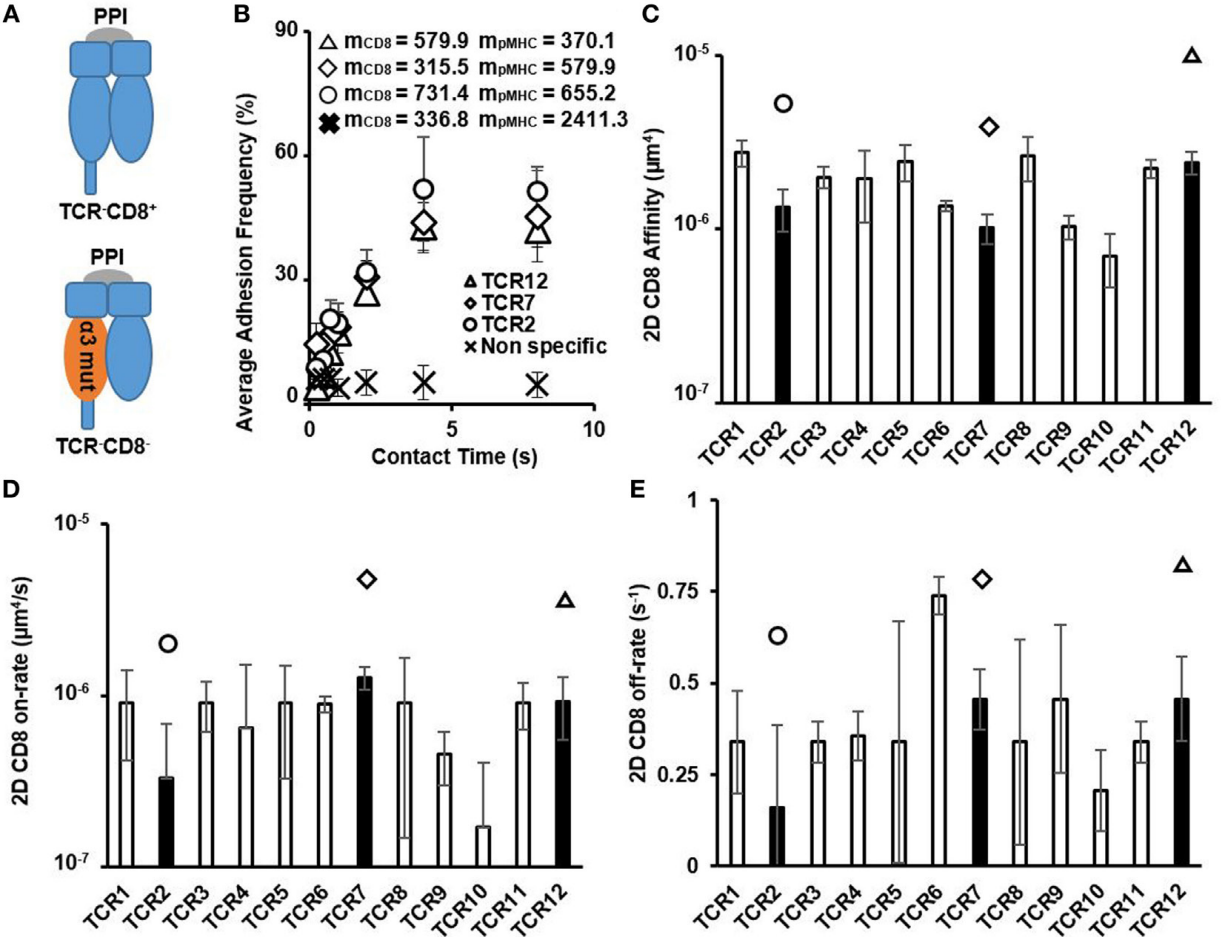
Two-dimensional (2D) CD8/pMHC affinity and kinetics of HCV-specific CD8+ T cell clones. **(A)** Schematics denoting the different peptides and MHC mutant used to isolate CD8/pMHC interactions and for non-specific interaction control. **(B)** Average adhesion frequency of three examples of 2D CD8 membrane kinetics of functional HCV-specific precursor CTLs show similar saturation contact times as 2D T cell receptor (TCR) membrane kinetics. Non-specific adhesion was measured on TCR12 and was represented as CD8 interaction with red blood cells conjugated with pMHC-CD8mut-preproinsulin (PPI) at same contact times as used in 2D CD8 affinity measurements. Values of m_CD8_ and m_pMHC_ were the total CD8 and pMHC site density (molecules/μm_2_) for each respective CTL when measurement was performed. **(C)** 2D CD8 affinity of all functional HCV-specific CTLs in order of ascending TCR affinity. 2D CD8 affinity was calculated using Eq. 3 in Section “Materials and Methods.” Filled bars represent the average 2D CD8. Affinity of the examples denoted in **(B)** with each filled bar having a symbol above it indicating individual CTLs. **(D)** 2D CD8 on-rate in order of ascending TCR affinity. 2D CD8 on- and off-rates were calculated using Eq. 2 in Section “Materials and Methods.” Filled bars denoting CTLs depicted in **(B)**. **(E)** 2D CD8 off-rate in order of ascending TCR affinity. 2D CD8 off-rate was calculated similarly to 2D TCR off-rate, using Chi-squared curve fitting of the 2D CD8 membrane kinetic curves for each CTL, respectively. Filled bars with symbols indicate individual CTLs depicted in **(B)**. All data shown as mean ± SD of at least three cell pairs.

We next interrogated the 2D affinity and kinetics of human CD8 interacting with pMHC by testing these HCV-specific CTLs interacting with PPI peptide in complex with pMHC (denoted as pMHC-CD8wt-PPI) (Figure 2A). Despite occupying a large range of 2D TCR affinity, the adhesion frequency curves of CD8 interacting with pMHC showed a consistent trend of approaching saturation with similar receptor and ligand site densities (Figure 2B). Measured 2D CD8 affinities were similar across different CTL clones and with respect to CD8αα:αβ ratio, total CD8 expression, and the change in temperature (Figures S4 and S10 in Supplementary Material). These human CD8 2D affinities were orders of magnitude smaller than the highest TCR affinity and were generally in a similar range to mouse CD8 affinity reported previously (Figure 2C; Table S1 in Supplementary Material) (19). The on- and off-rates were also comparable to what has been shown for mouse CD8 (Figures 2D,E; Table S1 in Supplementary Material) (19). Thus, human CD8 affinity alone should not vary significantly on different HLA alleles. We detected other TCRs that were of lower affinity compared to CD8 affinity in our previous study (8); however, these TCRs were not functional by cytotoxicity assay using APCs pulsed with either HCV or the irrelevant peptide PPI. This contrasts with our previous results using one mouse TCR interacting with a set of APLs (11), where several APLs had an even lower affinity compared to mouse CD8 yet still elicited functional responses.

### 2D TCR Affinity and On-Rate Correlate Better with T Cell Functionality Than the Off-Rate

Numerous studies have shown that 3D TCR off-rate negatively correlates with TCR activation potential, which ultimately links to T cell functionality (33–35). Compared to off-rate, on-rate was not deemed to have the same level of influence on TCR activation in the 3D setting. Two emerging 2D affinity analyses by both single molecule FRET and micropipette adhesion assay stressed the importance of 2D on-rate with respect to TCR activation, suggesting that increased on-rate may allow TCRs to more effectively scan APCs for pathogenic epitopes (4, 6). 2D on-rate also correlated better with T cell functionality compared with the 2D off-rate. However, these studies used one TCR interacting with a set of APLs. Thus, it is not known whether 2D on-rate can be of predictive value in polyclonal viral–antigen-specific T cells.

**Figure 3:**
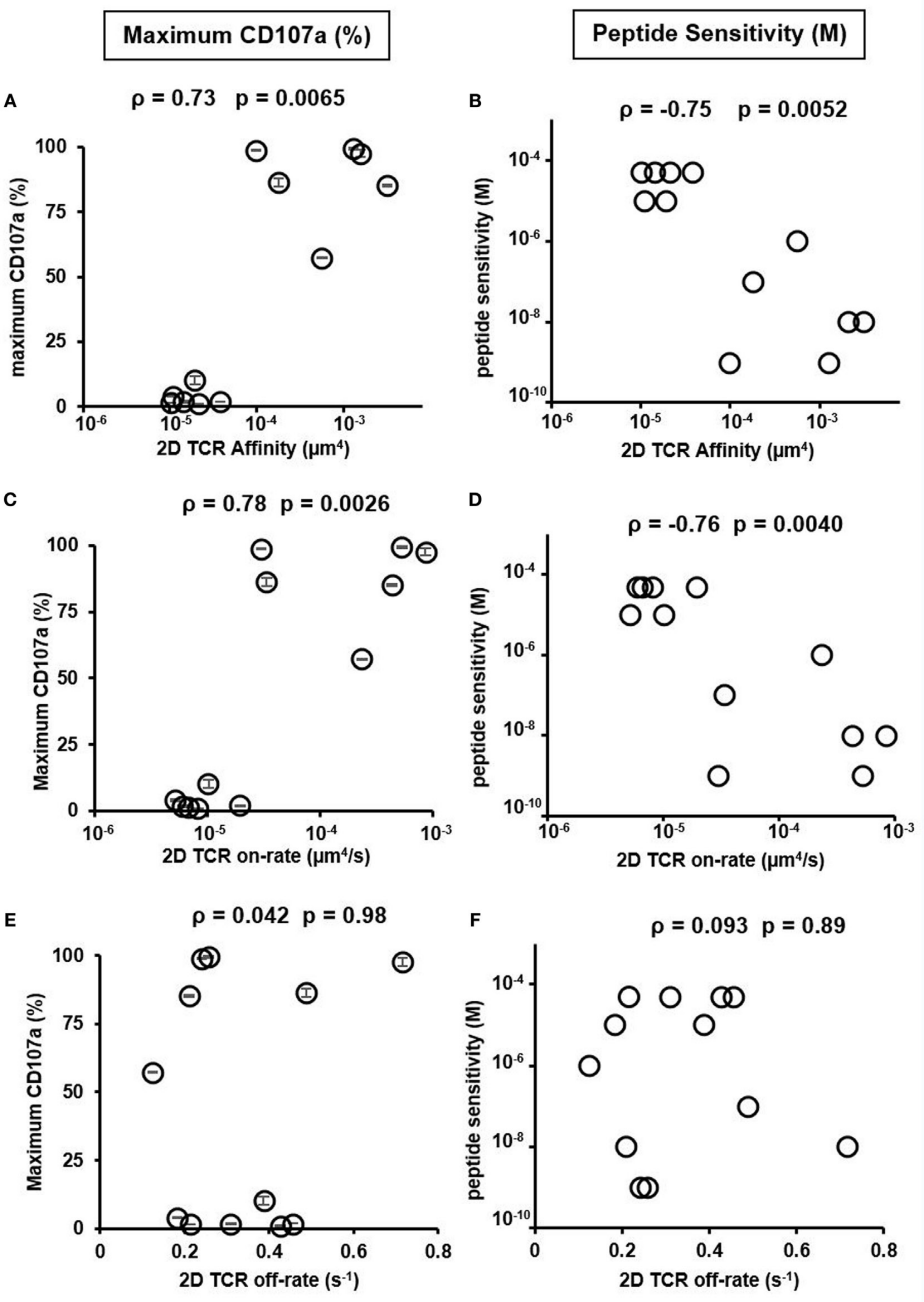
Two-dimensional (2D) T cell receptor (TCR) affinity and on-rate correlates with peptide sensitivity and functionality. **(A)** 2D TCR affinity versus maximum CD107a expression, defined as the CD107a positive T cell percentage after stimulation with the highest concentration of HCV peptide. **(B)** 2D TCR affinity versus peptide sensitivity, defined as the peptide concentration required to induce at least 1% CD107a positive T cells after subtracting background stimulation using irrelevant peptide, NY-ESO-1 (SLLMWITQV). **(C)** 2D TCR on-rate versus maximum CD107a expression, defined as in **(A)**. **(D)** 2D TCR on-rate versus peptide sensitivity, defined as in **(B)**. **(E)** 2D TCR off-rate versus maximum CD107a expression, defined as in **(A)**. **(F)** 2D TCR off-rate versus peptide sensitivity, defined as in **(B)**. All data points of CD107a were performed as duplicates and shown as the mean ± SD. The same result was obtained for the two peptide sensitivity experiments performed. Therefore, no error bar was shown in **(B,D,F)**. ρ and *p* values were determined by Spearman’s rank correlation.

We, therefore, used the set of previously isolated CTL clones to investigate the relationship between both the 2D on and off-rate with T cell functionality in the context of a set of polyclonal TCR interacting with the same ligand. We tested CTL functionality by two means: maximum CD107a expression, a marker for CTL degranulation after stimulation (17, 36), and peptide sensitivity (37), which quantifies the minimum peptide concentration required to stimulate CD107a^+^ T cells to a preset threshold. Previously CD107a expression was demonstrated in *ex vivo* stimulated human T cell clones to correlate well with specific lysis (36) and, therefore, is a good surrogate for cell killing capacity. A previous analysis of peptide potency showed similar correlation to 2D TCR affinity as in our study (8). Consistent with previous results (4, 6, 8), 2D TCR affinity and 2D TCR on-rate calculated at both room temperature and 37°C were highly correlated with the functionality of the HCV-specific clones (Figures 3A–D; Figure S11 in Supplementary Material), while 2D TCR off-rate was not (Figures 3E,F; Figure S11 in Supplementary Material). Examining three cytokines, IL-2, TNF-α, and INF-γ, on three CTL clones in high, medium, and low TCR affinity range yielded similar results as CD107a (Figure S8 in Supplementary Material). These data suggest that 2D affinity and on-rate are of greater predictive power for functionally competent cells, given the larger dynamic range of 2D affinity and on-rate compared to their 3D counter parts.

### TCRs That Are Independent of CD8 Cooperation Exist in the Human Antigen-Specific T Cell Repertoire

**Figure 4:**
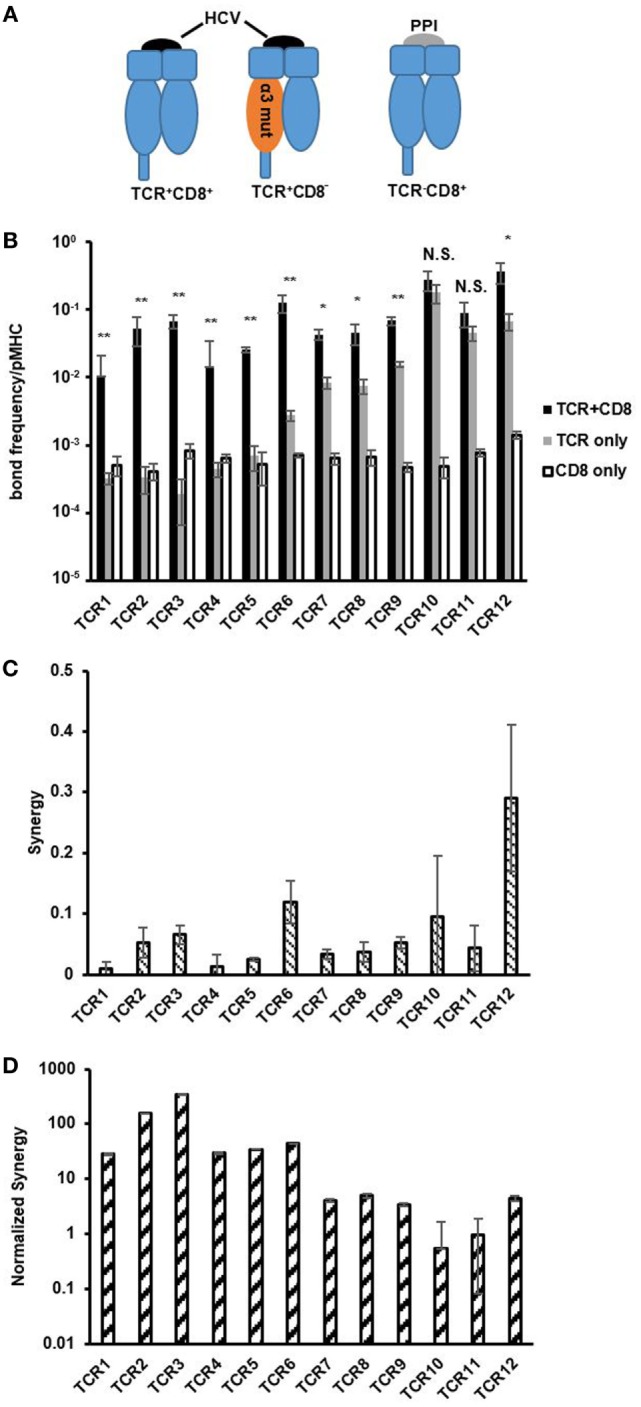
CD8 contribution decreases with increasing T cell receptor (TCR) affinity. **(A)** Schematics depicting pMHC variants used to isolate bimolecular TCR/pMHC and CD8/pMHC interaction and TCR/CD8/pMHC tri-molecular interaction. **(B)** Bond frequency per pMHC for TCR + CD8, TCR only, and CD8 only interactions with respective pMHCs for each of the functional CTLs in order of increasing two-dimensional (2D) TCR affinity. Bond frequency per pMHC was calculated following Eq. 4 in Section “Materials and Methods.” One-way ANOVA was performed between TCR + CD8 and TCR only bond frequency to assess statistical significance between values with *p*-values more than 0.05 considered not significant (NS), *p*-values less than 0.01 (*), and 0.001 (**) denoted by asterisk. **(C)** Synergy was calculated for each CTL in the traditional manner following Eq. 5 in Section “Materials and Methods,” and shown in order of increasing 2D TCR affinity. **(D)** Normalized synergy was calculated following Eq. 6 in Section “Materials and Methods,” and shown in order of increasing 2D TCR affinity. Data are shown as the mean ± SD of at least three cell pairs. Error propagation was performed to obtain SD for normalized synergy in **(D)**.

CD8 has been shown to increase the stability of the TCR/pMHC interaction by decelerating the dissociation of the receptors and reducing the overall off-rate of the reaction kinetics (9, 38, 39). Previous 3D affinity studies showed that there were varying degrees of CD8 cooperation – less cooperation with high-affinity TCR/pMHC interactions and more cooperation with low-affinity TCR/pMHC interactions using either one naturally occurring TCR interacting with a set of APLs (15) or a few protein engineered TCRs interacting with the same pMHC (14). However, later 2D affinity experiments showed that high-affinity TCR/pMHC interactions received more cooperation compared with their low-affinity counter parts (7, 11). Thus, it remains a challenge on how to address this discrepancy. Furthermore, it is not clear if naturally occurring antigen-specific polyclonal TCRs, with different orders of magnitude of affinity when interacting with the same pMHC, would have any cooperation between CD8 and TCR and if so, whether there are any naturally occurring TCRs that are independent of CD8 cooperation in binding. Using our set of HCV epitope-specific polyclonal CTLs and pMHC variants coupled with different epitopes (Figure 4A), we were able to systematically address these questions.

The large affinity differences between high- and low-affinity TCRs and between TCRs and CD8 make it difficult to compare their ligand binding propensity. To simplify this comparison, we converted 2D affinity to number of bonds formed per MHC molecule as previously described (4, 11). For TCR, this parameter also spanned a 500-fold range similar to 2D affinity (Figure 4B). By contrast, the number of bonds formed per MHC molecule for CD8 were generally in the same range as low-affinity TCRs and had little variation between TCRs where both TCR affinity and number of bonds formed per MHC molecule differed by orders of magnitude (Figure 4B). 37°C did not impact significantly on any of these measurements (Figure S12 in Supplementary Material).

The previously described 2D method of evaluating CD8 cooperation for 2D affinity analysis subtracted the number of bonds formed by TCR/pMHC and CD8/pMHC interactions from (TCR + CD8)/pMHC interactions, and was named synergy or the change in number of bonds (7, 11). In our data, we demonstrated a similar trend between synergy and 2D TCR affinity as has been seen previously in the 2D TCR kinetic data, which showed that high-affinity TCRs received more CD8 cooperation compared to low-affinity TCRs at both room temperature (Figure 4C) as well as 37°C (Figure S12 in Supplementary Material) (7, 11). However, there existed two high-affinity TCRs, TCR10, and TCR11, which had no statistically significant differences in the number of bonds formed per MHC molecule with and without CD8/pMHC binding, which conflicts with the results from synergy analysis that showed high-affinity TCRs in the 2D system received more CD8 help or cooperation than low-affinity TCRs (room temperature in Figure 4B and 37°C in Figure S12A in Supplementary Material). We hypothesized that the cooperation obtained as calculated by synergy was a total synergy that TCRs could receive, which was dependent on the total number of bonds that TCR/pMHC could form. Therefore, normalizing synergy by the number of TCR/pMHC bonds formed for each MHC molecule evaluates the synergy per TCR bond, which could be a more accurate parameter in the 2D setting to evaluate the cooperation between TCR and CD8.

Converting synergy to normalized synergy showed that low-affinity TCRs received more cooperation, or help, from CD8 per TCR bond compared to medium- and high-affinity TCRs at both room temperature (Figure 4D) and 37°C (Figures S12B,C in Supplementary Material). This corroborates the finding in previous 3D studies using protein engineered TCRs or APLs that higher affinity TCRs are more likely to be CD8 independent in binding than low-affinity TCRs (14, 15). Among the three high-affinity TCRs, two were essentially independent of CD8 in terms of pMHC binding. We also have functional data to support that these CD8 minimally dependent TCR expressing CLTs are more resistant to CD8 blocking and are able to retain most of the functionality in cognate peptide stimulation compared to TCRs that are more dependent on CD8 when most of CD8 molecules are blocked (below). Thus, our analysis using the new parameter, normalized synergy, was able to reconcile the discrepancy between previous 3D and 2D affinity analysis on CD8 cooperation to TCR/pMHC binding. Now, both 3D and 2D analyses show that high-affinity TCRs receive less help while low-affinity TCRs receive more help from CD8. It also revealed that it is possible to identify naturally occurring TCRs that are independent of CD8 in binding to pMHC, which would be beneficial for a range of applications, including adoptive T cell immunotherapies (40).

### Normalized Synergy Correlates Better with CTL Function Compared to Synergy

**Figure 5:**
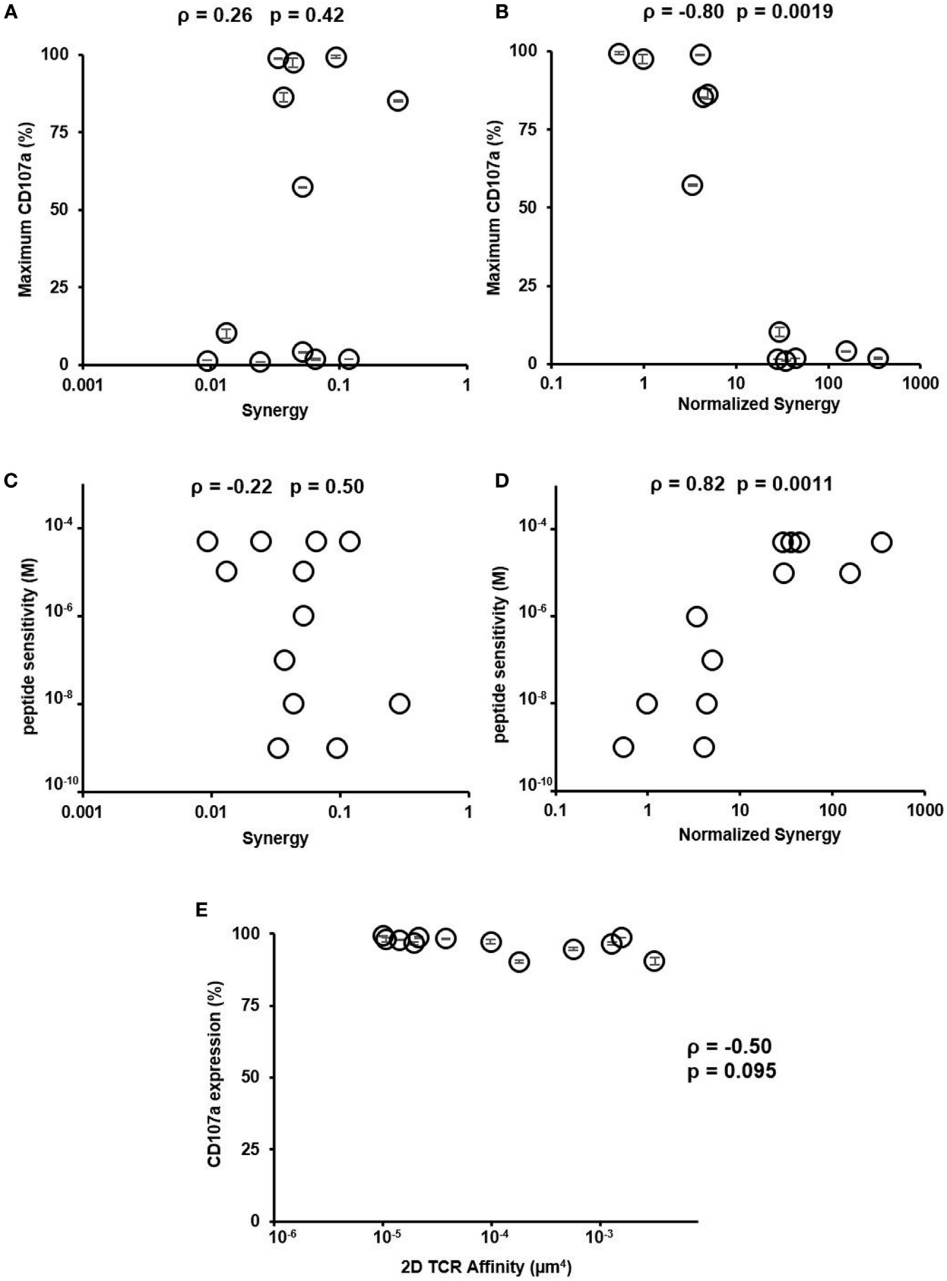
Normalized synergy correlates with functional output and peptide sensitivity. **(A)** Traditional synergy versus maximum CD107a expression, defined in Figure 3. **(B)** Normalized synergy versus maximum CD107a expression, defined in Figure 3. **(C)** Traditional synergy versus peptide sensitivity, defined in Figure 3. **(D)** Normalized synergy versus peptide sensitivity, defined in Figure 3. **(E)** Expression of CD107a on populations of functional HCV-specific CTLs after phorbol 12-myristate 13-acetate and ionomycin stimulation with respect to two-dimensional (2D) T cell receptor (TCR) affinity as measured by micropipette adhesion frequency assay. All data points for CD107a expression were shown as mean ± SD calculated from duplicates. The same results were obtained for the two peptide sensitivity experiments performed. Therefore, no error bar was shown in **(C,D)**. ρ and *p* values determined by Spearman’s rank correlation.

Having reconciled the discrepancy on how low- and high-affinity TCRs depend on CD8 in binding to pMHC, we next investigated if the newly characterized normalized synergy in 2D affinity analysis was predictive of CTL function. To accomplish this, we compared both synergy and normalized synergy to maximum CD107a expression or peptide sensitivity (Figure 5). Normalized synergy was shown to better correlate with both methods of evaluating CTL function compared to traditionally calculated synergy (Figures 5B,D). Using a smaller set of CTLs, we repeated TCR and CD8 affinity and kinetic measurement at 37°C. These data allowed us to calculate synergy and normalized synergy at 37°C and use them to correlate with CTL functional data. Consistent with the room temperature data, normalized synergy calculated at 37°C was better correlated with CTL functional data measured either by maximum CD107a^+^ T cells percentage or peptide sensitivity compared to traditional synergy (Figure S14 in Supplementary Material). The lack of correlation between traditionally calculated synergy and either functional parameters suggests that the total synergy that receptors could receive is an inaccurate assessment of the CD8 contribution to binding and has little predictive power of the functional response of a given TCR. To ensure that the functional response was not biased toward clones with inherently greater signaling capacity, we tested each of the CTLs functional response against PMA and ionomycin stimulation that bypass TCR signaling (41). This stimulation condition did not correlate with 2D TCR affinity, demonstrating that downstream signaling capacity was consistent between clones regardless of 2D TCR affinity (Figure 5E; Figure S8 in Supplementary Material). Overall, these results suggest that normalized synergy more accurately describes the cooperative effect of TCR and CD8 binding in the 2D system and it has more predictive power of the functional response of TCRs of varying 2D affinity to a single antigen compared to traditional synergy. Finally, the positive correlation between 2D TCR affinity and maximum CD107a (or the negative correlation between 2D TCR affinity and peptide sensitivity) (Figure 3) along with the negative correlation between maximum CD107a and normalized synergy (or positive correlation between peptide sensitivity and normalized synergy) (Figure 5) suggests that the higher the TCR affinity, the better the CTL functional response, and the less CD8 help each TCR/pMHC bond needs, which translates into the lower normalized synergy. Thus, using normalized synergy to characterize CD8 help in TCR/pMHC binding reconciles the discrepancy between previous 2D and 3D studies on CD8 contribution.

### CD8-Independent Clone Is Able to Maintain Functional Response in the Presence of CD8 Blocking Antibody

**Figure 6:**
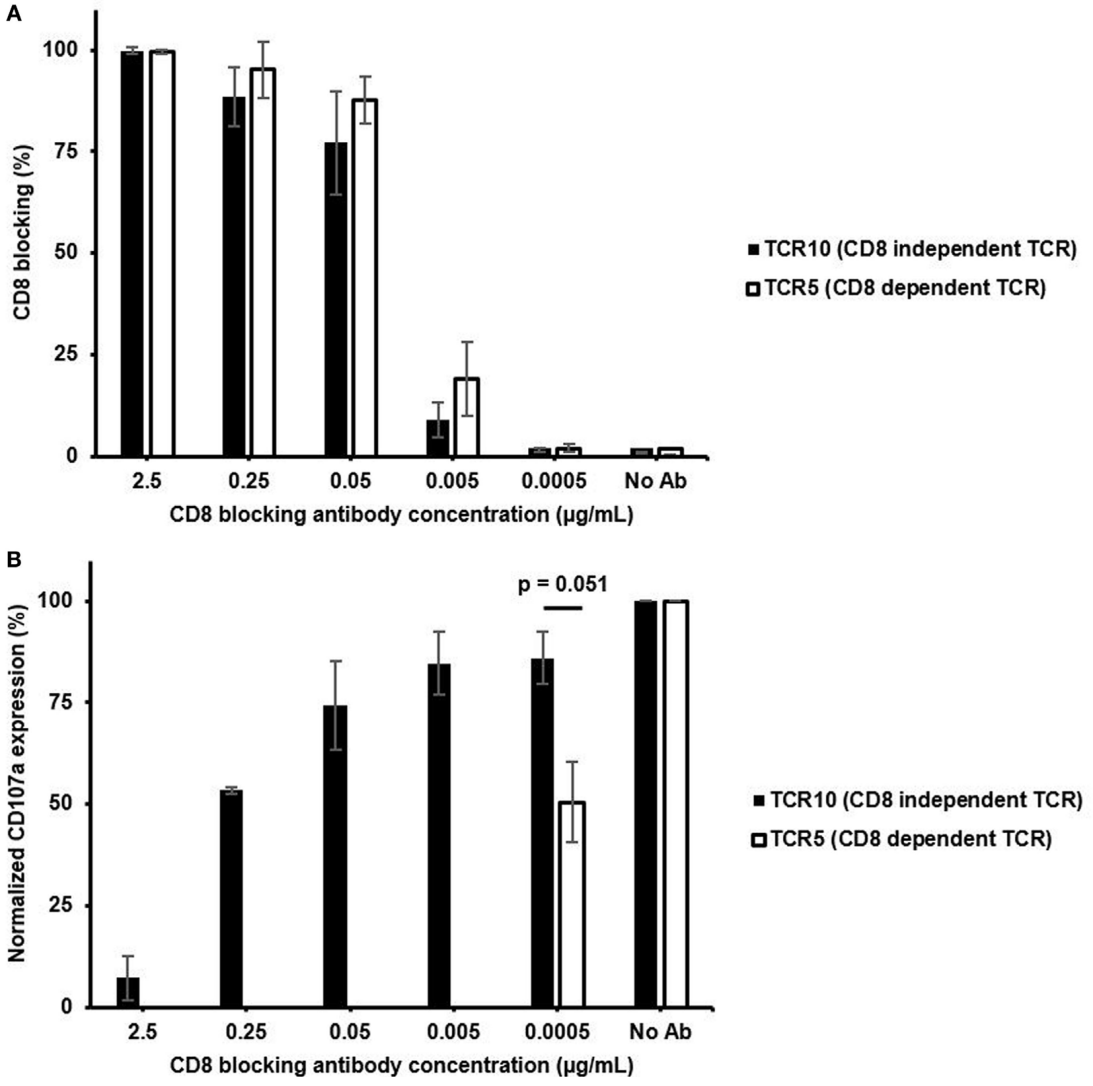
CD8-independent T cell receptor (TCR) is able to retain functional response in the presence of CD8 blocking antibody. **(A)** Percent of CD8 co-receptor that was blocked for each concentration of CD8 blocking antibody present during the 4 h of HCV peptide pulsed JY cell stimulation (Figure S6 in Supplementary Material). **(B)** Percentage of CD107a positive T cells stimulated with varying concentrations of CD8 blocking antibody for CD8-independent TCR (TCR10) and CD8-dependent TCR (TCR5). 10 µM of HCV peptide was presented throughout the stimulation. Percentage of CD107a positive T cells for each CD8 blocking condition was normalized to normal stimulation condition without CD8 blocking. Data in panel **(A,B)** were mean ± SD averaged from two independent experiments from two different days. One-way ANOVA was performed between normalized CD107a expression to assess statistical significance between values with *p*-value denoted above data.

To better understand the reliance of CD8 on the functional capacity of CD8-dependent or -independent TCR expressing CTLs, we performed a functional assay with varying degree of CD8 blocking during HCV peptide stimulation. The different concentration of CD8 blocking antibody was shown to block CD8 from 100% down to 1% for both CTLs (Figure 6A). Although the CD8-independent TCR clone lost about 20% functional response, even with as low as 1% of CD8 molecules being blocked, the CD8-dependent TCR clone suffered 50% of functional response under the same level of CD8 blocking (Figure 6B). For the CD8-independent TCR clone, it was not until more than 85% of CD8 molecules were blocked, which it lost 50% of the functional responses (Figure 6B). Thus, the CD8-independent clone was able to resist 500 times higher CD8 blocking antibody concentration compared to the CD8-dependent clone to retain 50% of functional response (ratio between two concentrations, at which both clones showed 50% of functional response on Figure 6B). A similar result was shown in the experiment where intracellular cytokine staining was examined as a way to evaluate T cell activation (Figure S8 in Supplementary Material). Without CD8 blocking even low-affinity TCR2 exhibited low amounts of cytokine positive cells (Figure S8A in Supplementary Material), while in the presence of CD8 blocking antibody, only CD8-independent TCR10 showed detectable levels of cytokine-producing cells (Figure S8B in Supplementary Material). Again, stimulation with PMA and ionomycin induced similar percentage of cytokine positive cells in all three CTL clones regardless of CD8 dependency and TCR affinity, suggesting that each CTL clone was capable of fully functional response if TCR signaling was bypassed (Figure S8C in Supplementary Material). Together, these results demonstrated that although CD8-independent TCR clone still requires some CD8 for full T cell function, it is able to retain a large percentage of T cell function even under a large range of CD8 density. Thus, these CD8-independent TCRs could be valuable to TCR re-directed adoptive T cell therapy where they can resist the differences on CD8 level in CD8^+^ T cells (20–22).

## Discussion

Using a set of viral epitope-specific polyclonal CTL clones, we performed a comprehensive analysis of 2D affinity and kinetics of human TCR and CD8 interacting with pMHC and investigated the synergistic effect between CD8 and TCRs with varying affinities. Our results confirmed the three orders of magnitude differences on polyclonal TCR affinities that we have previously observed (8) and the kinetics studied showed that these large differences in affinity stemmed from the equally large range of TCR 2D on-rate and small differences of TCR 2D off-rate (Figure 1). Both 2D affinity and on-rate highly correlated with CTL function as measured by maximum CD107a expression or peptide sensitivity (Figure 3). These results again validated the use of 2D affinity in searching for high-affinity TCRs with therapeutic potential for adoptive T cell immunotherapies (8).

As expected, the 2D affinities for CD8 were orders of magnitudes smaller compared to the highest affinity TCRs. This difference lies in the small 2D on-rate of CD8 as its 2D off-rate is in a comparable range as that of the TCR. For these CTL clones, 2D TCR affinities were larger than 2D CD8 affinity. This is because, here, we used functional CTL clones as defined previously (8, 36) by subtracting non-specific interactions with an irrelevant peptide pulsed APCs. This resulted in the functional clone 2D affinity threshold (8), which is 10^−5^ μm^4^. Compared to the 2D CD8 affinity, there were many more non-functional clones whose affinities were between CD8 affinity, 10^−6^ μm^4^, and the functional clone 2D TCR affinity threshold. These low-affinity T cells have been reported in mouse CD4^+^ (42) T cells as major responders for the primary immune response and in mouse CD8^+^ T cells (43). Although these low-affinity cells were enriched in tissue resident memory CD8^+^ T cells at a persistent infection phase, high-affinity T cells had a proliferative advantage in the acute infection (43). Thus, it is possible these low-affinity T cells play an alternative role in different stages of immunity. It is also possible that they may respond to other peptide stimulations with a higher affinity as suggested by a previous study using engineered TCRs (14).

Measuring the affinity of TCR and CD8 allowed us to systematically evaluate the contribution of CD8 to TCR/pMHC binding for TCRs of largely varying affinity. It has been shown that using engineered TCRs of extreme high-affinity can reach similar functional capacity independent of CD8 co-receptor. However, most TCRs of physiological affinity relied on CD8 help for binding to antigen as well as function, with higher affinity TCRs depending less on CD8 and lower affinity TCRs depending more on CD8. With the introduction of 2D affinity, a new parameter, synergy, was introduced to quantify the cooperation between CD8 and TCR. Two studies (7, 11), including one of our own, showed that CD8 contribution increased with increasing TCR affinity. However, this conclusion generated discrepancy with previous 3D TCR affinity studies (14, 15) where higher TCR affinity T cells were shown to be less dependent on CD8 on ligand binding. In this study, we re-evaluated this issue and discovered that synergy per TCR bond or normalized synergy, is a more accurate parameter in evaluating the CD8 dependency for each TCR/pMHC bond formed in the 2D system. We further showed that this normalized synergy is better correlated with CTL function than synergy estimated previously (7, 11).

In addition, we also showed that high-affinity TCRs that are independent of CD8 binding existed naturally. Two of the high-affinity TCRs we found are essentially independent of CD8 in pMHC binding. Although these TCR expressing CTL clones still required CD8 to fully execute cytotoxicity (Figure S5 in Supplementary Material), they are much better in resisting CD8 expression level changes compared to CD8-dependent TCR expressing CTL clones (Figure 6). Thus, expressing these TCRs on adoptively transferred T cells would allow them to resist natural difference on CD8 expression level and still function (20–22). Given these new findings, our previously developed *in situ* TCR measurement method iTAST could be updated to measure normalized synergy, which would speed up the search for high-affinity, CD8-independent TCRs for therapeutic applications.

In summary, we have shown that the T cells recognizing a naturally occurring viral peptide span an affinity range of three orders of magnitude, a range that is predominantly due to their 2D on-rate. In our interrogation of the CD8 contribution, we established a new parameter to evaluate cooperation that can be attributed to CD8 and demonstrated that this normalized synergy correlates well with maximal functionality and peptide sensitivity. Finally, we demonstrated the presence of naturally occurring viral-specific CD8^+^ T cells in the circulating repertoire of HCV seronegative donors which do not rely upon CD8 for cognate ligand binding. These CD8-independent T cells have increased functionality and sensitivity compared to those more dependent upon CD8 for binding. In the future, CD8-independent TCRs might prove beneficial for the field of adoptive cell transfer for cancer immunotherapy because these TCRs are highly sensitive and functional in response to antigen stimulation in comparison to TCRs that more heavily relied on CD8 contribution for TCR/pMHC binding. In conclusion, utilizing these TCRs with less CD8 contribution might be beneficial for genetically engineered adoptive T cell immunotherapy techniques by eliciting greater functional capacities, possibly yielding better outcomes in clinical applications.

## Author Contributions

CW and AS performed all experiments; CW designed experiments and did all analyses; S-QZ, KM, and CH helped with CTL clones; TY, SE, and CK helped with experiment design; NJ conceived the idea, designed the study, directed data analysis, and wrote the paper with contributions from all coauthors.

## Conflict of Interest Statement

All other authors declare that the research was conducted in the absence of any commercial or financial relationships that could be construed as a potential conflict of interest NJ is a scientific advisor of ImmuDX, LLC.
