# Supplementary material for: Normalized Synergy Predicts That CD8 Co-Receptor Contribution to T Cell Receptor (TCR) and pMHC Binding Decreases As TCR Affinity Increases in Human Viral-Specific T Cells
Source: Front Immunol. 2017 Jul 28;8:894. doi: 10.3389/fimmu.2017.00894 (PMC5532383; doi:10.3389/fimmu.2017.00894)
Supplement: Supplementary file 1 [file Data_Sheet_1.PDF]

Supplementary Material

Normalized synergy predicts that CD8 co-receptor contribution to TCR and pMHC binding decreases as TCR affinity increases in human viral-specific T cells

Chad M. Williams[1], Alexandra A. Schonnesen[1], Shu-Qi Zhang[2], Ke-Yue Ma[3], Chenfeng He[1], Tori Yamamoto[4,5], S. Gail Eckhardt[6], Christopher A. Klebanoff[7,8], Ning Jiang[1,3,$]

Authors and Affiliations:

[1]Department of Biomedical Engineering, University of Texas at Austin, Austin, TX 78712, USA

[2]McKetta Department of Chemical Engineering, University of Texas at Austin, Austin, TX 78712, USA

[3]Institute for Cell and Molecular Biology, University of Texas at Austin, Austin, TX 78712, USA

[4]Center for Cancer Research, National Cancer Institute, National Institutes of Health, Bethesda, MD 20982, USA

[5]Immunology Graduate Group, University of Pennsylvania, Philadelphia, PA 19104, USA.

[6]LIVESTRONG Cancer Institutes, Dell Medical School, The University of Texas at Austin, Austin, TX 78712, USA

[7]Center for Cell Engineering and Department of Medicine, Memorial Sloan Kettering Cancer Center (MSKCC), New York, NY 10065, USA

[8]Parker Institute for Cancer Immunotherapy, MSKCC, New York, NY 10065, USA

[$]Correspondence should be addressed to:
Ning Jiang, Ph.D.
Email: jiang@austin.utexas.edu
Phone: 512-471-4860

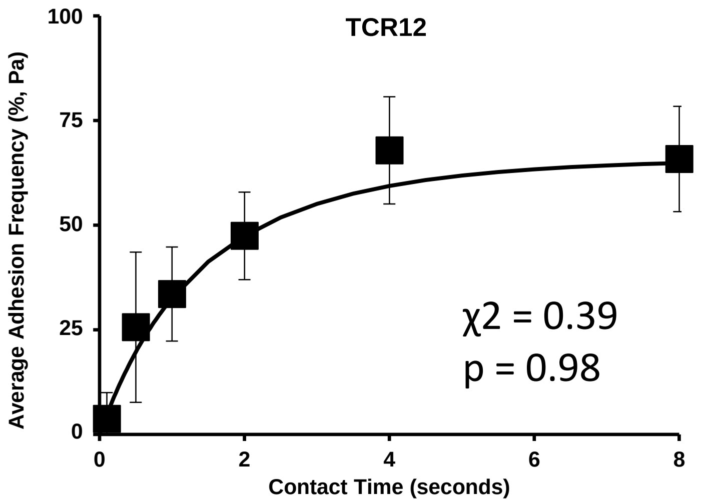

Figure S1. Chi-squared fitting of 2D off-rate for TCR/pMHC interaction
Chi-squared fit of adhesion curve of TCR binding to pMHC-CD8mut-HCV was used to determine 2D TCR off-rates. Then 2D TCR off-rate was used in combination with 2D TCR affinity to calculate the 2D TCR on-rate using equation 2. CD8 off-rate and on-rate were obtained using the same method. All data shown as mean ± S.D. (standard deviation) of at least 3 cell pairs.

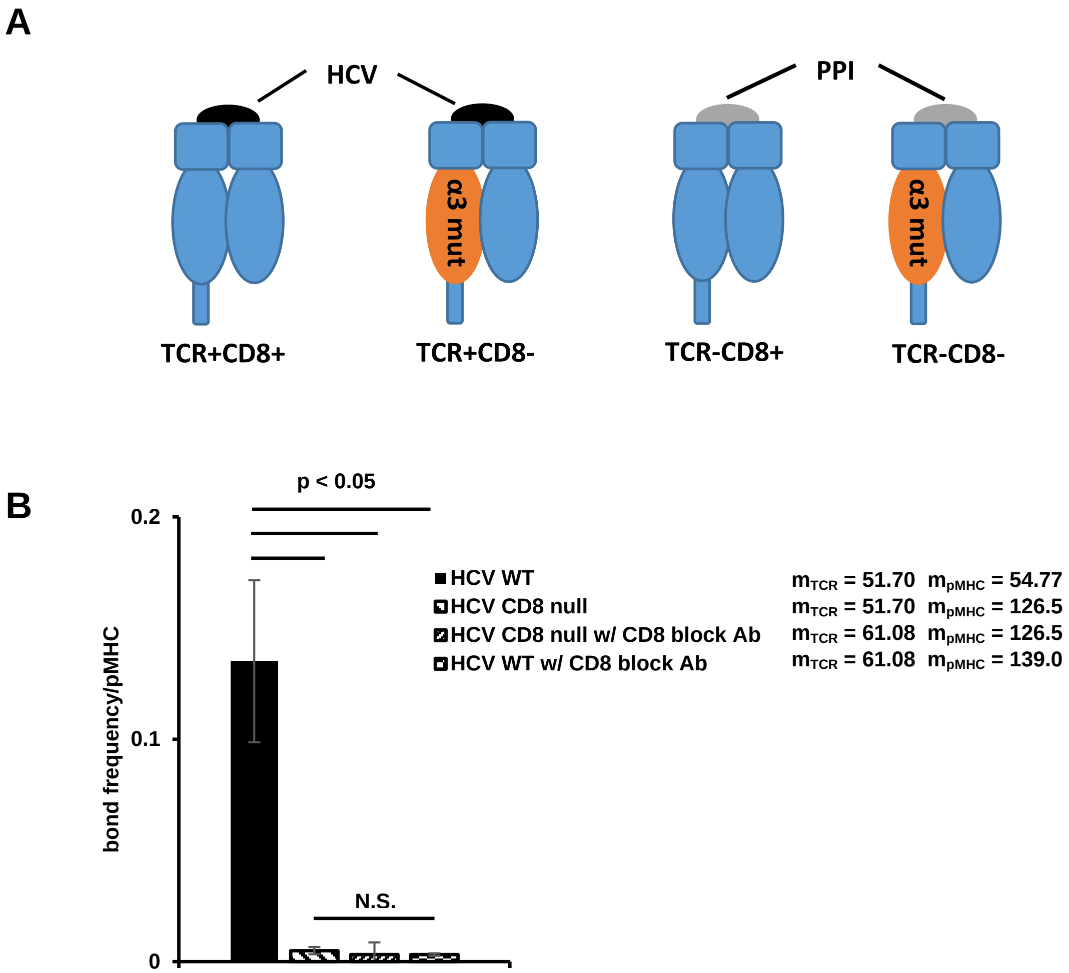

Figure S2. CD8 blocking antibody effectively eliminates CD8 contribution to pMHC binding

(A) Schematic depicting different pMHC variants and peptides used to isolate bimolecular TCR/pMHC and CD8/pMHC interaction and TCR/CD8/pMHC tri-molecular interaction. **(B)** Bond frequency/pMHC showed drastic difference between bond frequencies associated with pMHC-CD8wt-HCV monomer and pMHC-CD8mut-HCV monomer without CD8 blocking antibody, yet no difference was observed with CD8 receptor blocking. Values of m_{TCR} and m_{pMHC} are the TCR and pMHC site density when measurement was performed. All data shown as mean ± S.D. of at least 3 cell pairs. Student's T-test was performed on data sets corresponding to the different conditions with a p-value greater than 0.05 considered not significant (N.S.).

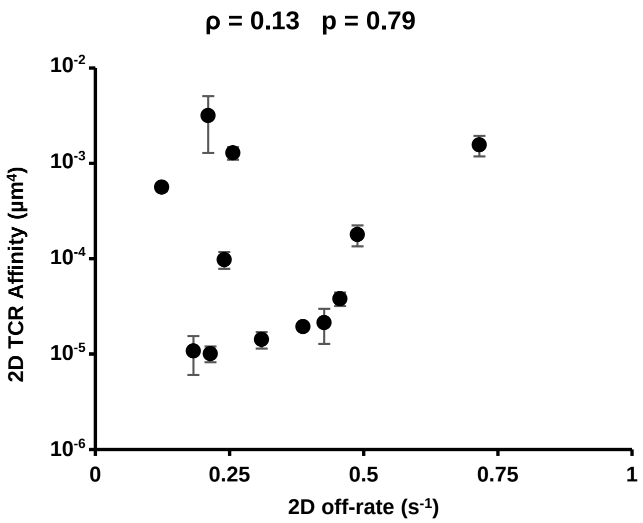

Figure S3. 2D TCR affinity and 2D off-rate correlation

ρ and p-value between 2D TCR affinity and off-rate was calculated using Spearman correlation. All data on 2D TCR affinity shown as mean ± S.D. of at least 3 cell pairs as described in **Figure 1**.

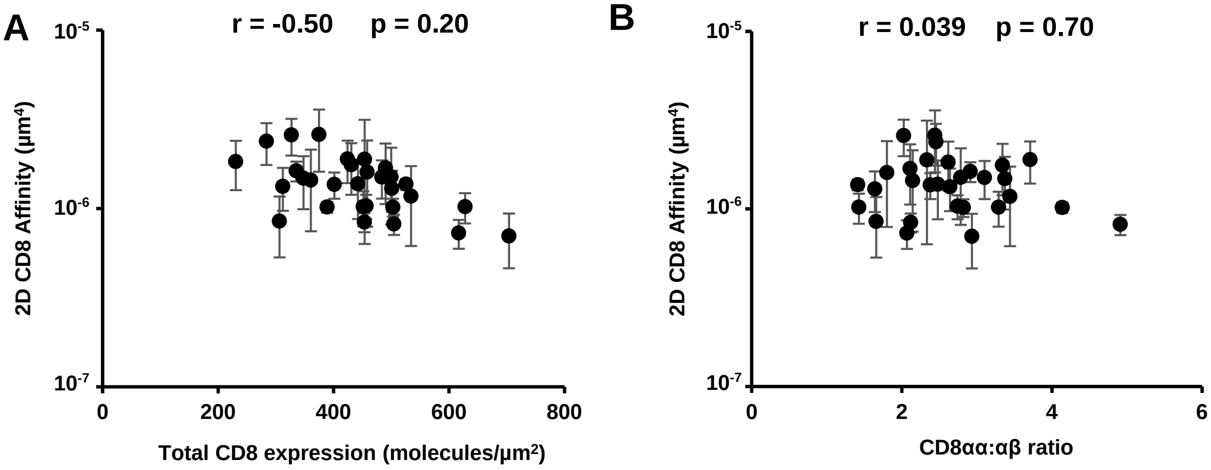

Figure S4. 2D TCR affinity in relationship with total CD8 expression and CD8αα:αβ ratio **(A)** 2D TCR affinity versus total CD8 expression. CD8α and CD8β expression was determined by PE-labeled antibody specific to either CD8α or CD8β chain. CD8αβ expression was calculated by CD8β expression level. Then CD8αα was calculated by following formula, (CD8α - CD8β)/2, and total expression was determined by adding CD8αα and CD8αβ expression together. **(B)** 2D TCR affinity versus CD8αα and CD8αβ expression ratio. CD8αα and CD8αβ expression ratio was calculated using the CD8αα and CD8αβ expression level defined in (**A**) for all CTL clones. All data are shown as mean ± S.D. of at least 3 cell pairs. r and p values were determined by Pearson correlation.

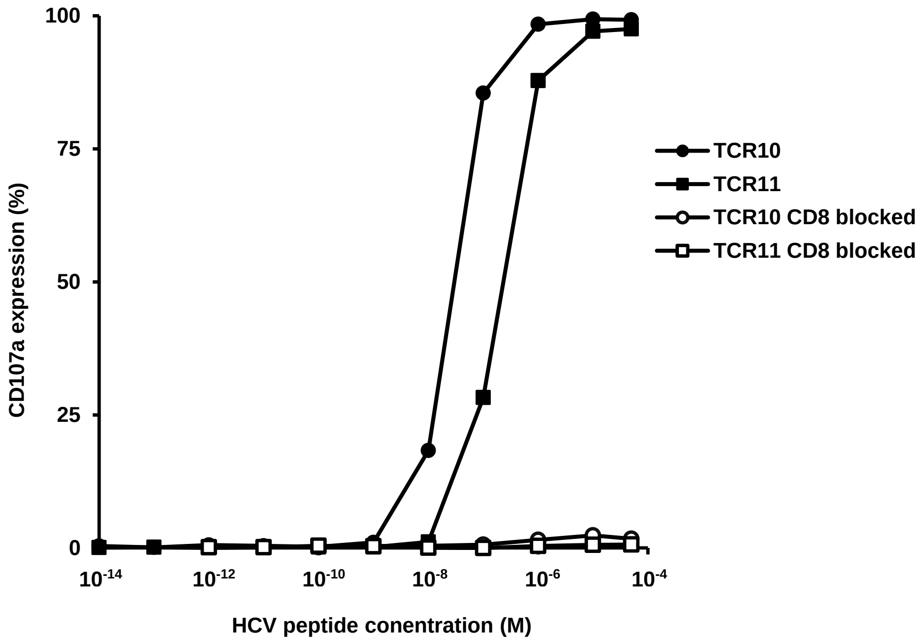

Figure S5. CD8 independent TCRs in pMHC binding show need for CD8 in eliciting functional response

CD107a expression level was assessed for two CTL clones, where TCR binding to pMHC was determined independent of CD8, in the presence or absence of CD8 blocking antibody. All data on HCV peptide stimulation with and without CD8 blocking antibody were performed as duplicates and are shown as an average ± S.D.

A

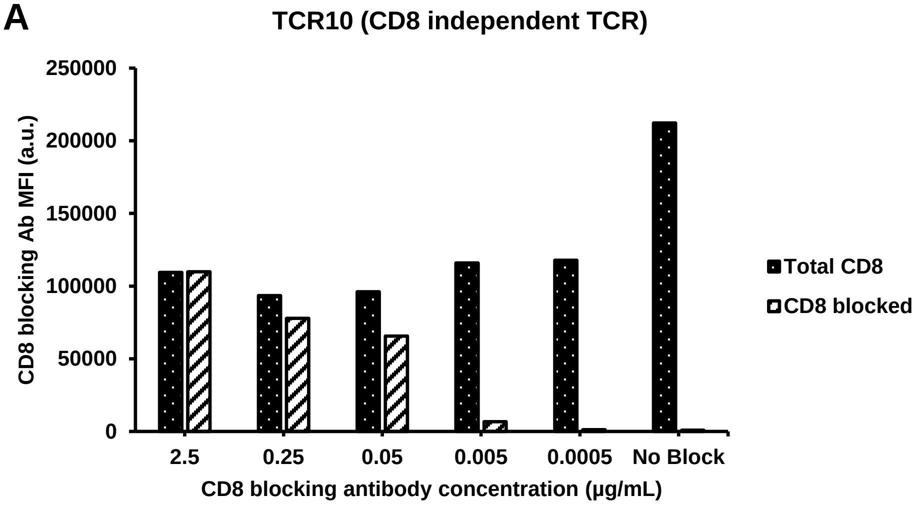
TCR10 (CD8 independent TCR)
CD8 blocking Ab MFI (a.u.)
250000
200000
150000
100000
50000
0
2.5
0.25
0.05
0.005
0.0005
No Block
CD8 blocking antibody concentration (µg/mL)
Total CD8
CD8 blocked

B

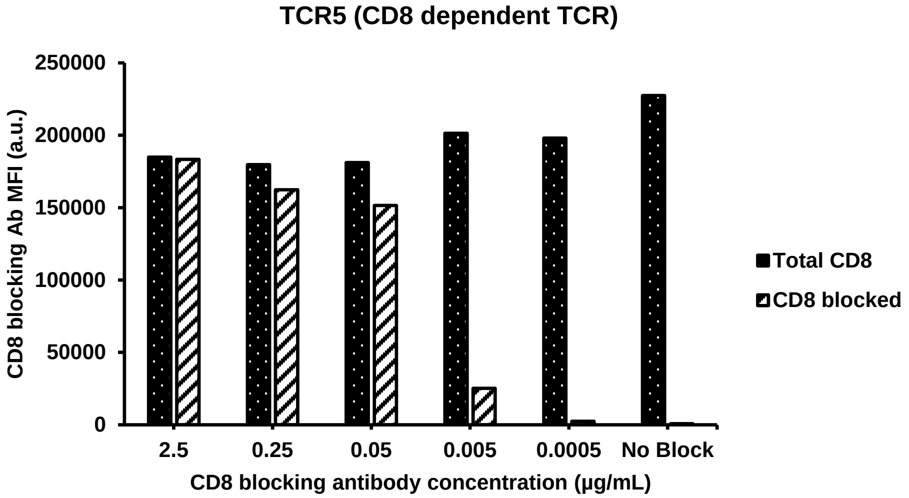
TCR5 (CD8 dependent TCR)
CD8 blocking Ab MFI (a.u.)
250000
200000
150000
100000
50000
0
2.5
0.25
0.05
0.005
0.0005
No Block
CD8 blocking antibody concentration (µg/mL)
Total CD8
CD8 blocked

C

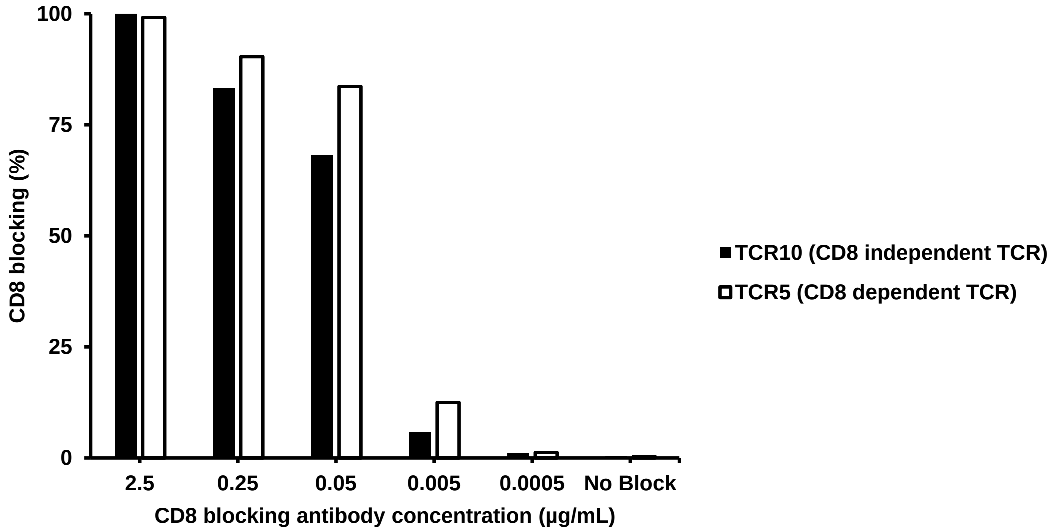
CD8 blocking (%)
100
75
50
25
0
2.5
0.25
0.05
0.005
0.0005
No Block
CD8 blocking antibody concentration (µg/mL)
TCR10 (CD8 independent TCR)
TCR5 (CD8 dependent TCR)

Figure S6. CD8 blocking under various blocking antibody concentrations for CD8 independent and dependent TCR expressing CTL clones.

(A) Mean fluorescence intensity (MFI) of CD8 blocking antibody for the CD8 independent TCR10 with additional saturating CD8 blocking antibody (filled, measuring total CD8 level) or without additional saturating CD8 blocking antibody (stripe, measuring blocked CD8 level) during 10µM HCV peptide stimulation. Throughout 4 hour stimulation with 10µM HCV peptide, CD8 blocking antibody was present at various concentrations. At the end of 4 hour stimulation cells were split into two populations. One was stained with additional saturating CD8 blocking antibody for 30 minutes, and the other was left without this addition. MFI generated from additional CD8 blocking antibody and the original CD8 blocking antibody added at the beginning of the assay denoted total CD8 expression (Total CD8), while MFI generated from the original CD8 blocking antibody added at the beginning of the assay denoted the portion of CD8 being blocked. **(B)** Mean fluorescence intensity (MFI) of CD8 blocking antibody for the CD8 dependent TCR5 with additional saturating CD8 blocking antibody (filled, measuring total CD8 level) or without additional saturating CD8 blocking antibody (stripe, measuring blocked CD8 level) during 10µM HCV peptide stimulation. **(C)** Ratio between MFI of CD8 blocked and total CD8 multiplied by 100 denote percentage of CD8 receptor blocked during stimulation due to varying concentration of CD8 blocking antibody for both TCR5 and TCR10. All CD8 blocking antibody MFI represented as arbitrary units (a.u.). Two independent experiments from two separate days were performed. Results from one representative experiment were shown here.

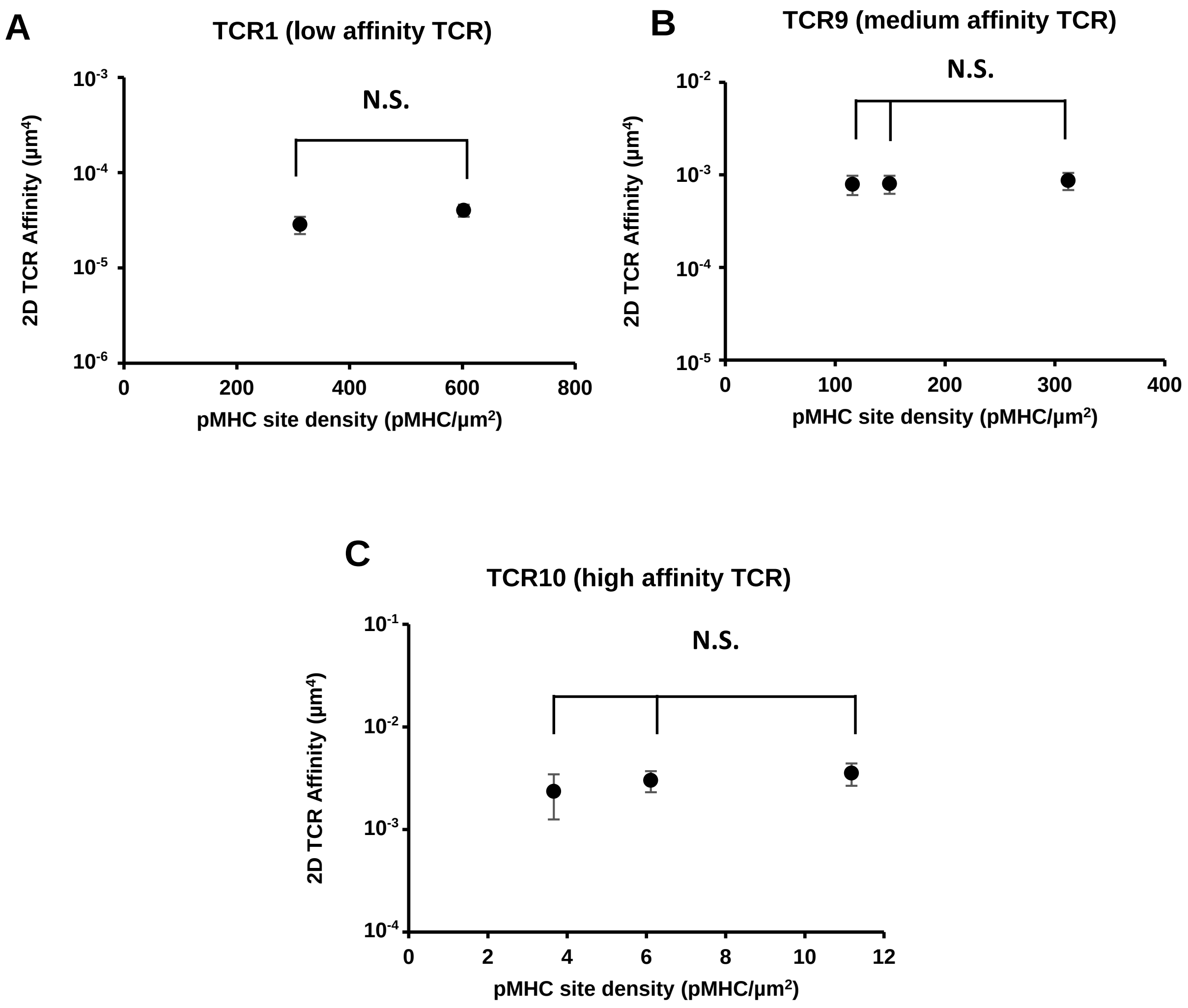

Figure S7. Relationship between 2D TCR affinity and varying pMHC site densities
(A) Relationship between 2D TCR affinity with varying pMHC site densities for one low affinity TCR. **(B)** Relationship between 2D TCR affinity with varying pMHC site densities for one medium affinity TCR. **(C)** Relationship between 2D TCR affinity with varying pMHC site densities for one high affinity TCR. Pairwise ANOVA used to calculate significant differences between the 2D TCR affinities calculated at different pMHC site densities. A p-value more than 0.05 considered Not Significant (N.S.).

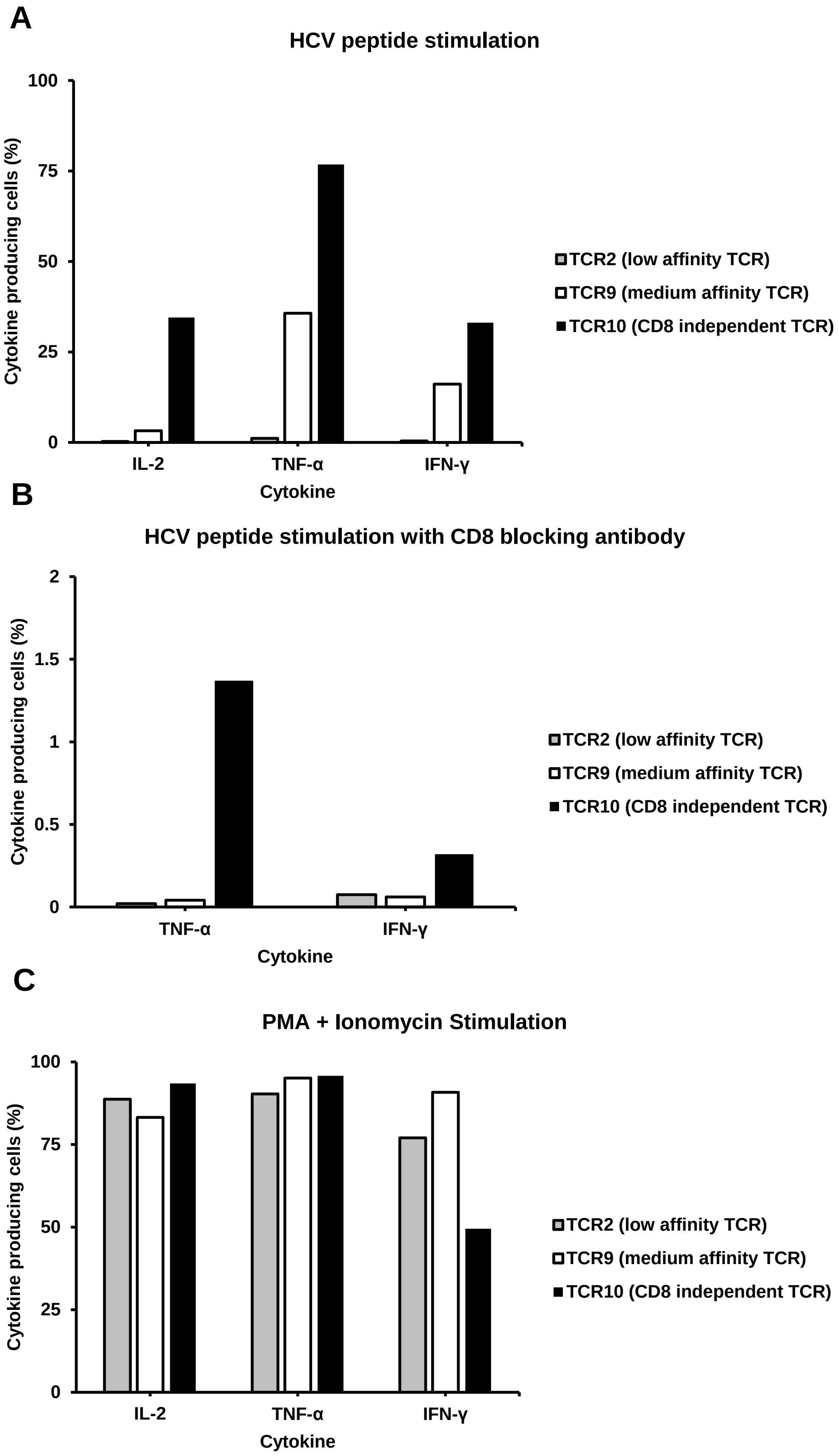
A
HCV peptide stimulation
Cytokine producing cells (%)
100
75
50
25
0
IL-2
TNF-α
IFN-γ
Cytokine
TCR2 (low affinity TCR)
TCR9 (medium affinity TCR)
TCR10 (CD8 independent TCR)
B
HCV peptide stimulation with CD8 blocking antibody
Cytokine producing cells (%)
2
1.5
1
0.5
0
TNF-α
IFN-γ
Cytokine
TCR2 (low affinity TCR)
TCR9 (medium affinity TCR)
TCR10 (CD8 independent TCR)
C
PMA + Ionomycin Stimulation
Cytokine producing cells (%)
100
75
50
25
0
IL-2
TNF-α
IFN-γ
Cytokine
TCR2 (low affinity TCR)
TCR9 (medium affinity TCR)
TCR10 (CD8 independent TCR)

Figure S8. Intracellular Cytokine Production due to various stimulation conditions
(A) Percentage of cells producing intracellular cytokines IL-2, TNF-α, and IFN-γ due to 10µM HCV peptide stimulation. HCV peptide stimulation was performed with 50,000 T cells from each CTL stimulated with 10µM HCV peptide in cell media at 37°C for 5 hour in the presence of brefeldin A and monensin to inhibit release of cytokine upon activation. After 5 hours cells were fixed and permeabilized, then stained with anti-human IL-2, TNF-α, and IFN-γ specific antibodies for 30 minutes. After 30 minutes stained cells were washed twice and analyzed by flow cytometry. This methodology was performed for all stimulation conditions. Cytokine production assay was performed on low affinity TCR (TCR2), medium affinity TCR (TCR9), and CD8 independent TCR (TCR10). **(B)** Percentage of cells producing intracellular cytokines TNF-α and IFN-γ due to 10µM HCV peptide stimulation in the presence of 2.5µg/mL CD8 blocking antibody. This concentration of CD8 blocking antibody was shown to block 100% of the CD8 co-receptor on the surface of the cell in CD107a CD8 blocking experiment (Figure S6 in Supplementary Material). **(C)** Percentage of cells producing intracellular cytokines IL-2, TNF-α, and IFN-γ due to PMA + Ionomycin stimulation. To quantify the percentage of cells expressing intracellular cytokine for all stimulation conditions, irrelevant peptide stimulation with 10µM NY-ESO-1 peptide was used for gating out non-specific cytokine production.

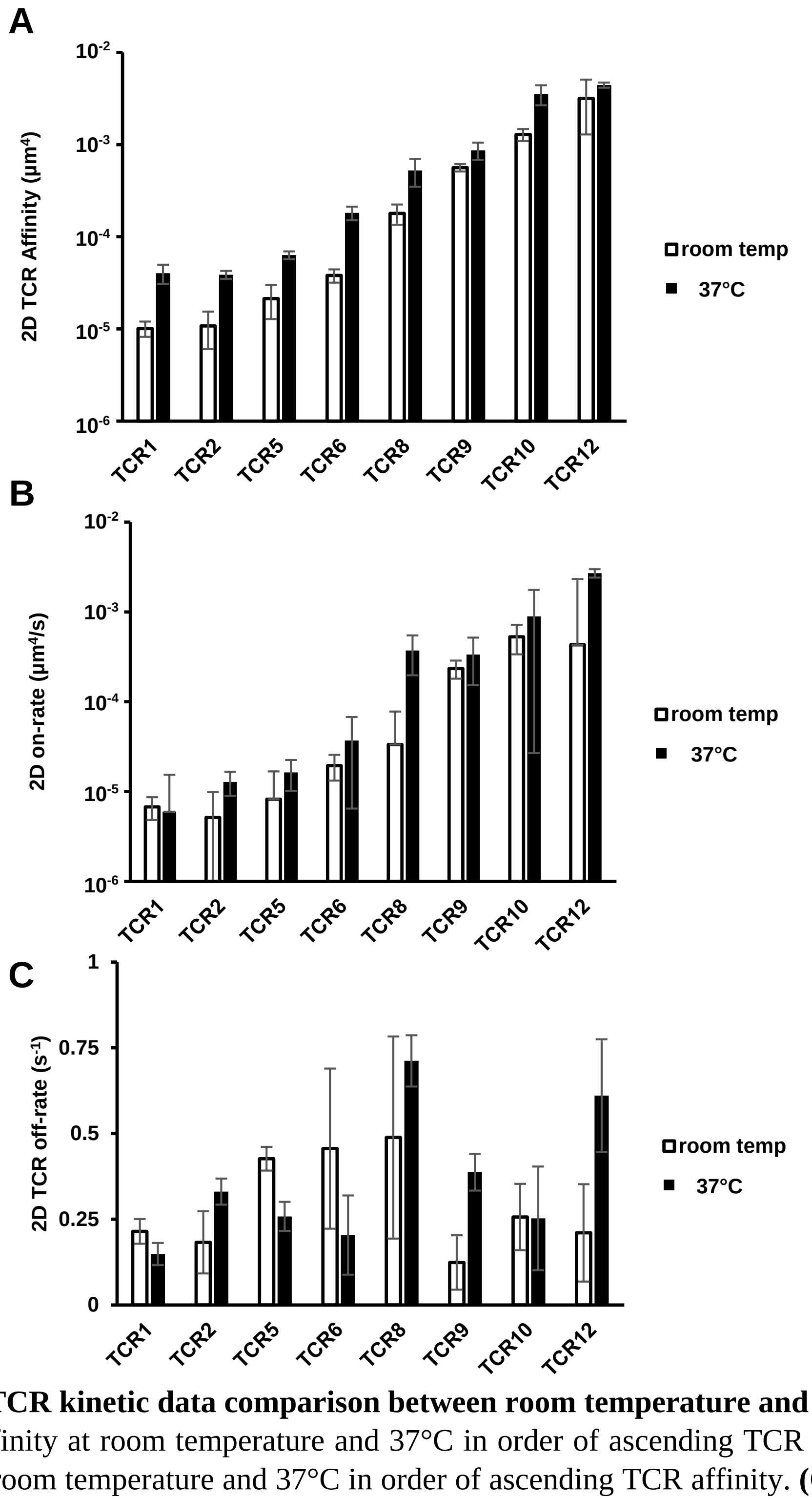

Figure S9. 2D TCR kinetic data comparison between room temperature and 37°C
(A) 2D TCR affinity at room temperature and 37°C in order of ascending TCR affinity. **(B)** 2D TCR on-rate at room temperature and 37°C in order of ascending TCR affinity. **(C)** 2D TCR off-rate at room temperature and 37°C in order of ascending TCR affinity. All data shown as mean ± S.D. of at least 3 cell pairs for both temperature conditions.

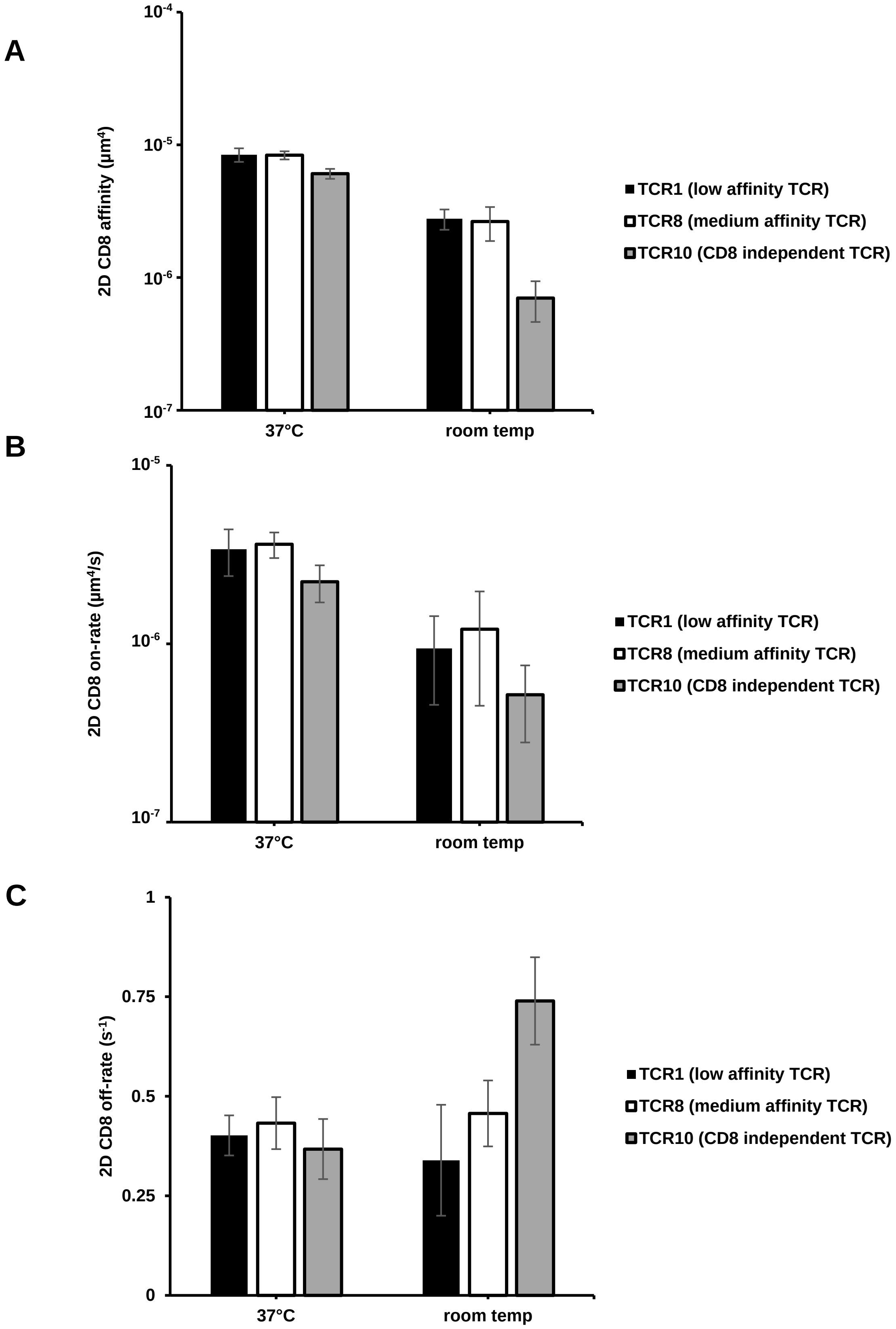
A
2D CD8 affinity (μm⁴)
10-4
10-5
10-6
10-7
37°C
room temp
TCR1 (low affinity TCR)
TCR8 (medium affinity TCR)
TCR10 (CD8 independent TCR)
B
2D CD8 on-rate (μm⁴/s)
10-5
10-6
10-7
37°C
room temp
TCR1 (low affinity TCR)
TCR8 (medium affinity TCR)
TCR10 (CD8 independent TCR)
C
2D CD8 off-rate (s-1)
1
0.75
0.5
0.25
0
37°C
room temp
TCR1 (low affinity TCR)
TCR8 (medium affinity TCR)
TCR10 (CD8 independent TCR)

Figure S10. 2D CD8 kinetic data comparison between room temperature and 37°C

(A) 2D CD8 affinity at room temperature and 37°C for three representative TCRs. **(B)** 2D CD8 on-rate at room temperature and 37°C for three representative TCRs. **(C)** 2D CD8 off-rate at room temperature and 37°C for three representative TCRs. All data shown as mean ± S.D. of at least 3 cell pairs for both temperature conditions.

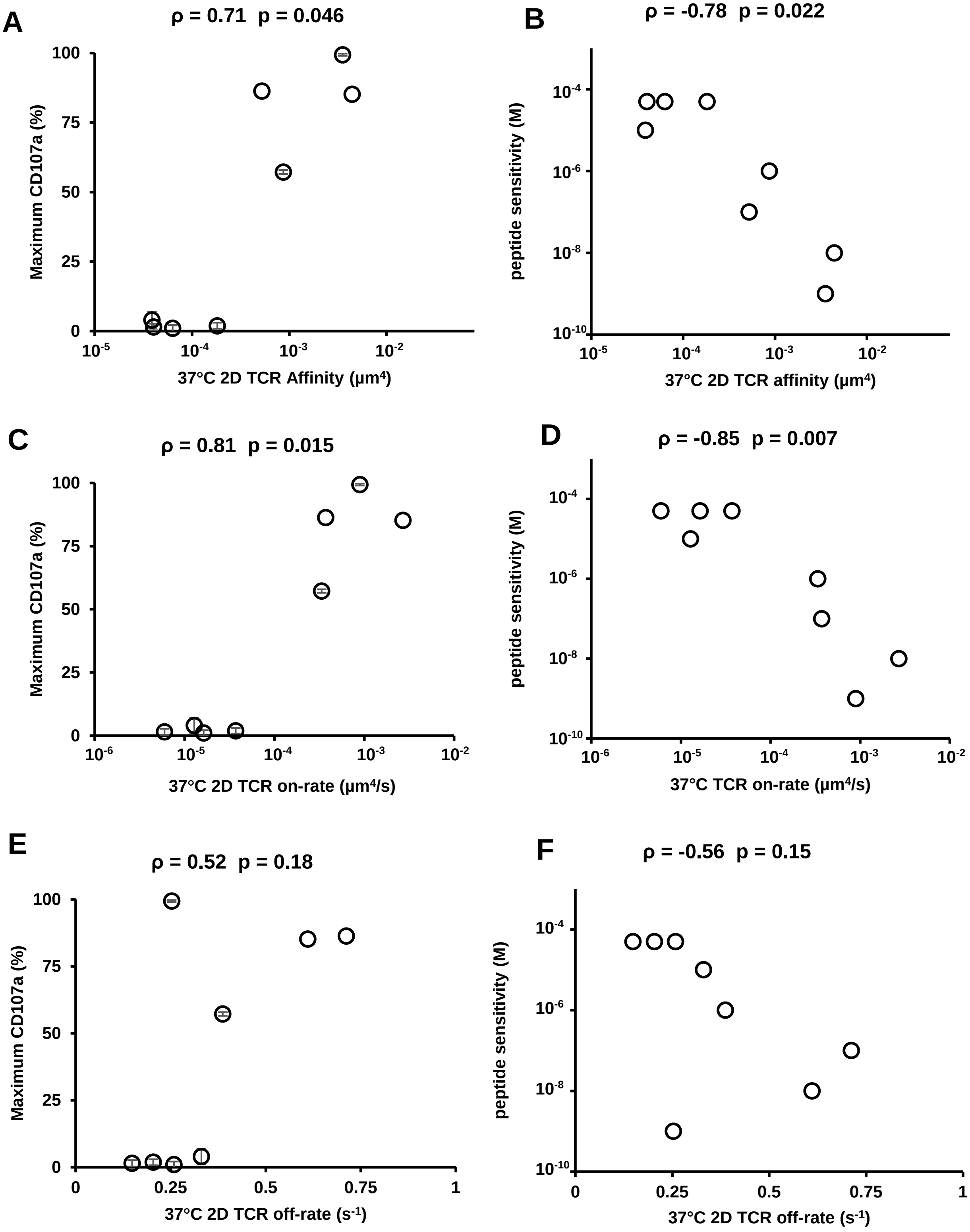
A
ρ = 0.71 p = 0.046
Maximum CD107a (%)
37°C 2D TCR Affinity (μm⁴)
B
ρ = -0.78 p = 0.022
peptide sensitivity (M)
37°C 2D TCR affinity (μm⁴)
C
ρ = 0.81 p = 0.015
Maximum CD107a (%)
37°C 2D TCR on-rate (μm⁴/s)
D
ρ = -0.85 p = 0.007
peptide sensitivity (M)
37°C TCR on-rate (μm⁴/s)
E
ρ = 0.52 p = 0.18
Maximum CD107a (%)
37°C 2D TCR off-rate (s⁻¹)
F
ρ = -0.56 p = 0.15
peptide sensitivity (M)
37°C 2D TCR off-rate (s⁻¹)

Figure S11. Relationship of 2D TCR kinetic data at 37°C and CTL functional response
(A) 2D TCR affinity at 37°C versus maximum CD107a expression. **(B)** 2D TCR affinity at 37°C versus peptide sensitivity. **(C)** 2D TCR on-rate at 37°C versus maximum CD107a expression. **(D)** 2D TCR on-rate at 37°C versus peptide sensitivity. **(E, F)** 2D TCR off-rate at 37°C versus maximum CD107a expression and peptide sensitivity. All data points of CD107a were performed as duplicates and are shown as the mean ± S.D. ρ and p values were determined by Spearman's rank correlation.

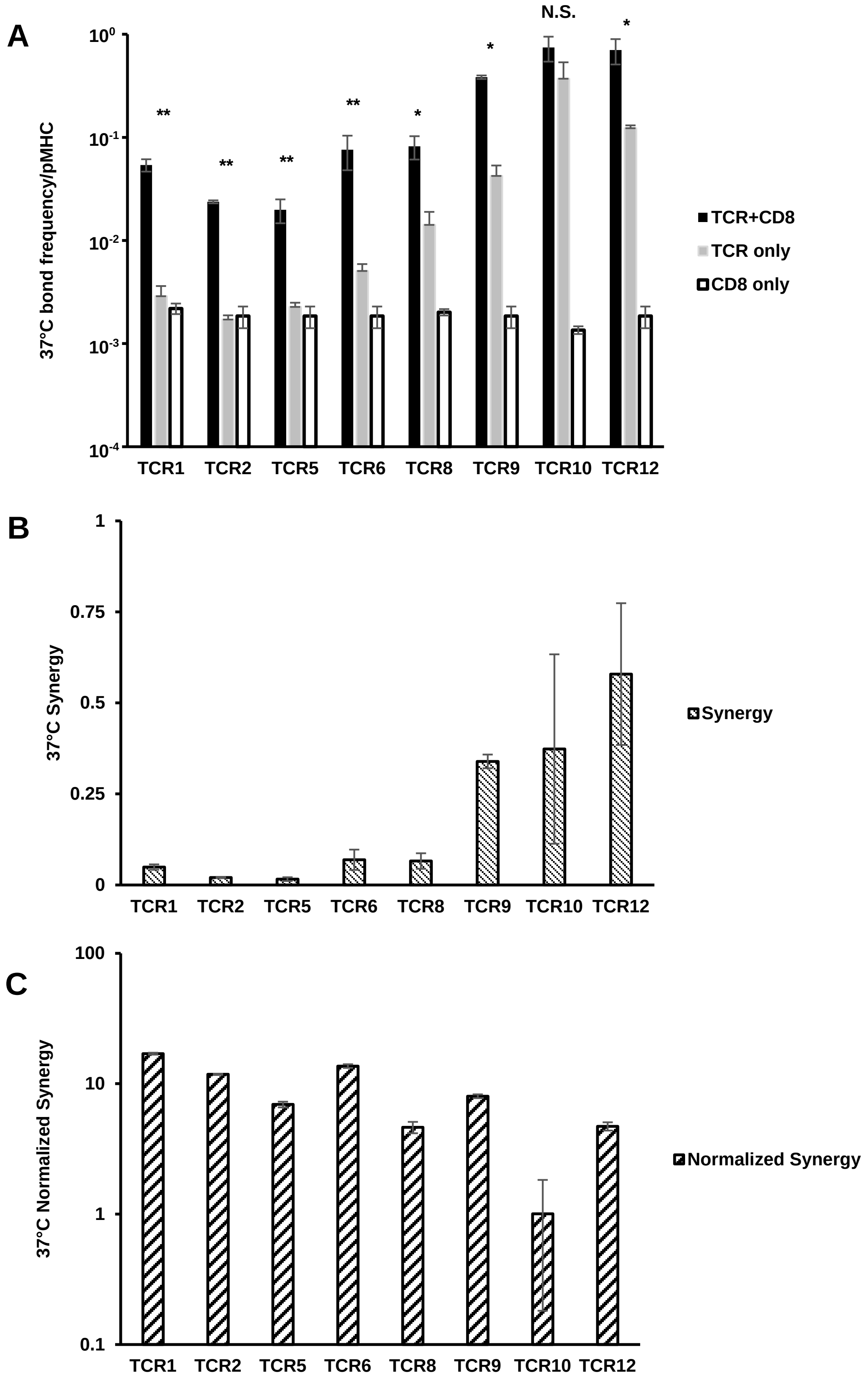
A
N.S.
10^{0}
10^{-1}
10^{-2}
10^{-3}
10^{-4}
37°C bond frequency/pMHC
TCR1 TCR2 TCR5 TCR6 TCR8 TCR9 TCR10 TCR12
TCR+CD8
TCR only
CD8 only
B
37°C Synergy
1
0.75
0.5
0.25
0
TCR1 TCR2 TCR5 TCR6 TCR8 TCR9 TCR10 TCR12
Synergy
C
37°C Normalized Synergy
100
10
1
0.1
TCR1 TCR2 TCR5 TCR6 TCR8 TCR9 TCR10 TCR12
Normalized Synergy

Figure S12. Comparison of CD8 contribution to TCR clones between room temperature and 37°C

(A) Bond frequency per pMHC for TCR+CD8, TCR only, and CD8 only interactions with respective pMHCs for each of the functional CTLs in order of increasing 2D TCR affinity at 37°C. One-way ANOVA was performed between TCR+CD8 and TCR only bond frequency to assess statistical significance between values with p-values more than 0.05 considered not significant (N.S.), p-values less than 0.01 (*) and 0.001 (**) denoted by asterisk. **(B)** Synergy was calculated for each CTL in the traditional manner following **equation 5** in materials and methods, and shown in order of increasing 2D TCR affinity at 37°C. **(C)** Normalized synergy shown in order of increasing 2D TCR affinity at 37°C. Data are shown as the mean ± S.D. of at least 3 cell pairs. Error propagation was performed to obtain S.D. for normalized synergy.

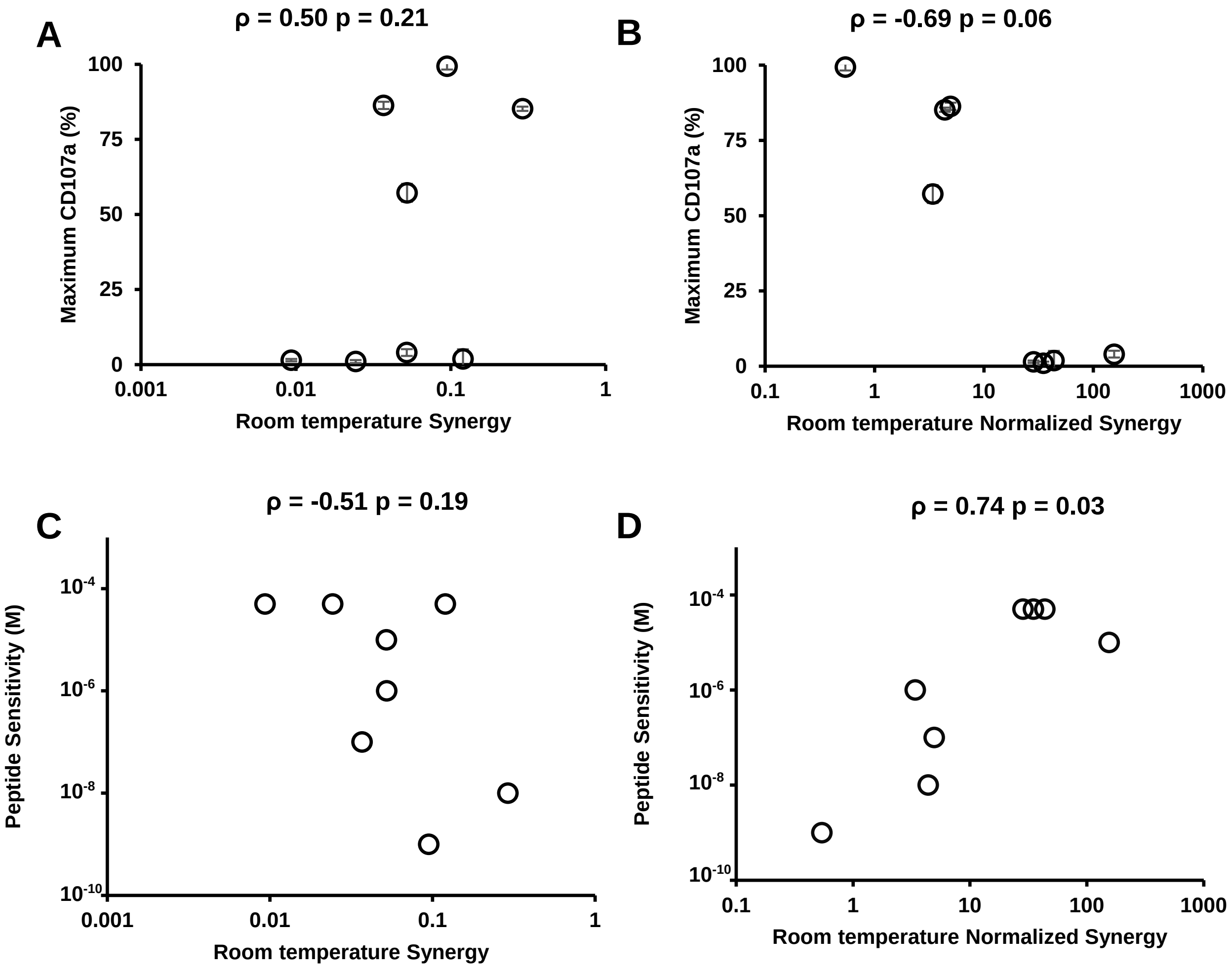

Figure S13. Room temperature synergy and normalized synergy vs functional response of a smaller set of TCR clones used for 37°C **measurements**

(A) Synergy correlation with maximum CD107a expression. **(B)** Normalized synergy versus peptide sensitivity. **(C)** Synergy versus peptide sensitivity. **(D)** Normalized synergy versus peptide sensitivity. All data points of CD107a were performed as duplicates and are shown as the mean ± S.D. ρ and p values were determined by Spearman's rank correlation.

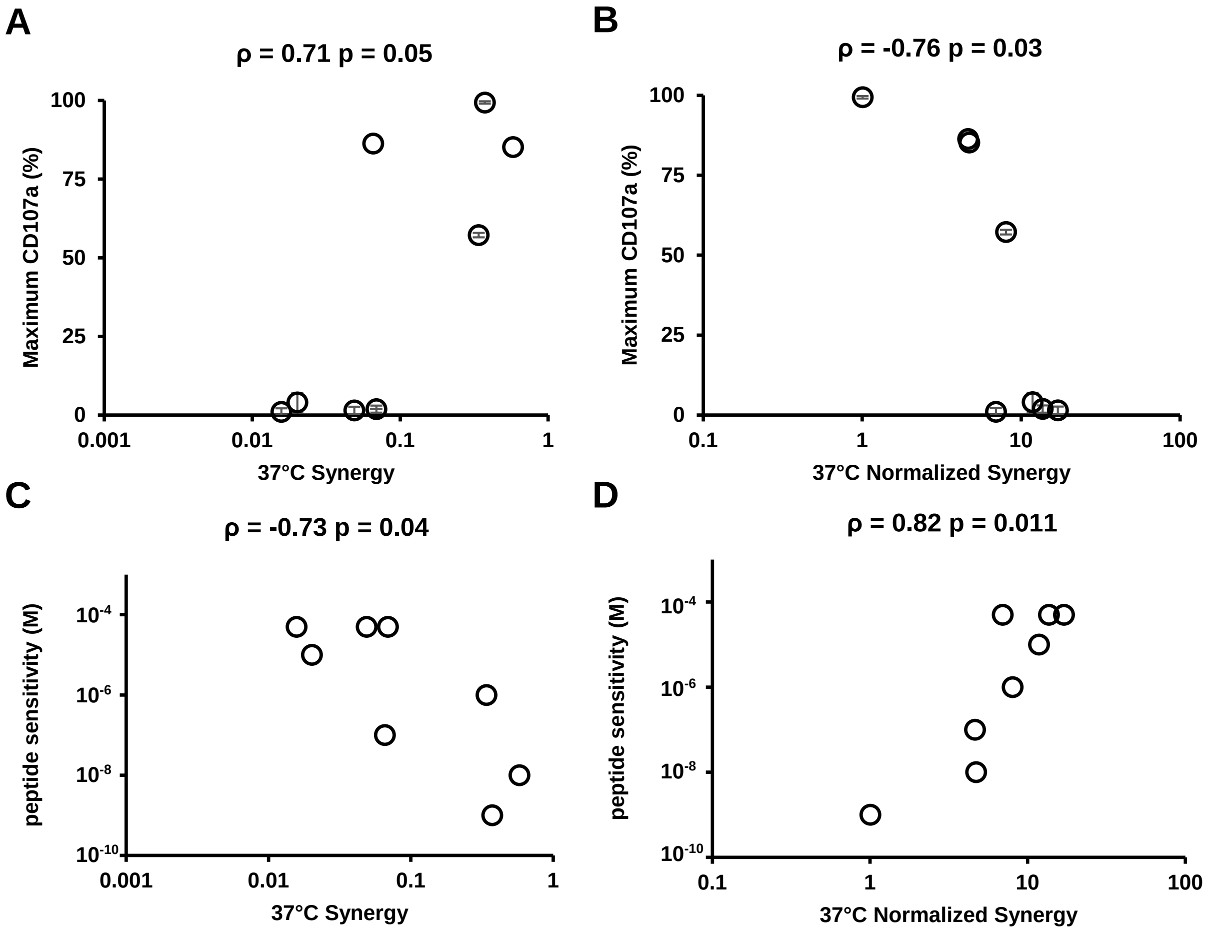

Figure S14. Synergy and normalized synergy vs functional response for a smaller set of CTL clones measured at 37°C

(A) Synergy versus maximum CD107a expression at 37°C. **(B)** Normalized Synergy versus peptide sensitivity at 37°C. **(C)** Synergy versus peptide sensitivity at 37°C. **(D)** Normalized synergy versus peptide sensitivity at 37°C. All data points of CD107a were performed as duplicates and are shown as the mean ± S.D. ρ and p values were determined by Spearman's rank correlation.

Supplementary Table

Table T1. Summary of 2D CD8 affinity, on-rate, and off-rate differences between mouse and human 2D CD8/pMHC measurements.

CD8 coreceptor	**Mouse**	**Human**
2D Affinity (μm^4) ± S.D.	$5.31x10^{-6} \pm 1.62x10^{-6}$	$1.65x10^{-6} \pm 6.67x10^{-7}$
2D on-rate ($\mu m^4/s$) ± S.D.	$6.39x10^{-6} \pm 3.08x10^{-6}$	$7.06x10^{-7} \pm 3.09x10^{-7}$
2D off-rate (s^{-1}) ± S.D.	1.16 ± 0.35	0.402 ± 0.146
